# Targeted Metabolomic Assessment of the Sub-Lethal Toxicity of Halogenated Acetic Acids (HAAs) to *Daphnia magna*

**DOI:** 10.3390/metabo11020100

**Published:** 2021-02-10

**Authors:** Lisa M. Labine, Myrna J. Simpson

**Affiliations:** 1 Department of Chemistry, University of Toronto, 80 St. George St., Toronto, ON M5S 3H6, Canada; lisa.labine@mail.utoronto.ca; 2 Environmental NMR Centre and Department of Physical and Environmental Sciences, University of Toronto Scarborough, 1265 Military Trail, Toronto, ON M1C 1A4, Canada

**Keywords:** ecotoxicology, disinfection by-products, dichloroacetic acid (DCAA), trichloroacetic acid (TCAA), dibromoacetic acid (DBAA)

## Abstract

Halogenated acetic acids (HAAs) are amongst the most frequently detected disinfection by-products in aquatic environments. Despite this, little is known about their toxicity, especially at the molecular level. The model organism *Daphnia magna*, which is an indicator species for freshwater ecosystems, was exposed to sub-lethal concentrations of dichloroacetic acid (DCAA), trichloroacetic acid (TCAA) and dibromoacetic acid (DBAA) for 48 h. Polar metabolites extracted from *Daphnia* were analyzed using liquid chromatography hyphened to a triple quadrupole mass spectrometer (LC-MS/MS). Multivariate analyses identified shifts in the metabolic profile with exposure and pathway analysis was used to identify which metabolites and associated pathways were disrupted. Exposure to all three HAAs led to significant downregulation in the nucleosides: adenosine, guanosine and inosine. Pathway analyses identified perturbations in the citric acid cycle and the purine metabolism pathways. Interestingly, chlorinated and brominated acetic acids demonstrated similar modes of action after sub-lethal acute exposure, suggesting that HAAs cause a contaminant class-based response which is independent of the type or number of halogens. As such, the identified metabolites that responded to acute HAA exposure may serve as suitable bioindicators for freshwater monitoring programs.

## 1. Introduction

Disinfection by-products (DBPs) are ubiquitous in aquatic ecosystems, and, to date, approximately 700 DBPs have been identified and detected [1]. DBPs are generated through chemical reactions between residual contaminants and organic matter found in effluents during wastewater treatment [1,2,3]. Chlorination, chloramination, chlorine dioxide and other halogenated disinfection methods are subject to high energies, which in turn lead to secondary product formation [4,5]. Previous work has shown that residual organic matter in the water and the implementation of UV irradiation led to an increase in the formation of DBPs such as trihalomethanes (THMs), haloacetic acids (HAAs), cyanogen chloride and chloroform [3,6]. Some of the most widely detected DBPs in drinking water and polluted source water include: chloroform, halogenated acetic acids (HAAs) and trihalomethanes [7,8]. In addition to being found in contaminated waters, the HAAs dichloroacetic acid (DCAA) and trichloroacetic acid (TCAA) can also be produced as metabolites of certain halogenated solvents and reactants (trichloroethylene and perchloroethylene) used in industrial and military applications [9,10,11]. TCAA and DCAA are also used in dermal cosmetic procedures, including the removal of warts [12,13]. HAAs exhibit high water solubility and low Henry’s law constants [14,15] and can persist in aquatic environments. Because HAAs form during the disinfection of wastewater into drinking water, it is likely that their concentrations in aquatic ecosystems will increase [16], especially in areas receiving effluents. Some HAAs may degrade in the environment depending on the presence of dehalogenation enzymes present in specific microbes [17]. Additionally, some HAAs may undergo oxidative dehalogenation, dehalogenation by methyl transfer and substitutive dehalogenation [18]. It is important to note that these processes are more prevalent in monohalogenated acetic acids [19], making dihalogenated and trihalogenated acetic acids more persistent due to their inherent chemistry. For example, TCAA has exhibited a high stability and persistence in both field and laboratory studies [17,19,20]. While dihalogenated acetic acid, such as DCAA, are more susceptible to some dehalogenation mechanisms than their trihalogenated counterparts [19], they are present at concentrations which exceed those of other HAAs present in aquatic environmental samples [20]. Individual HAAs have been reported in the low µg/L concentration range, but, collectively, the concentration of all HAAs can be significantly higher [21,22]. While many studies have assessed the environmental persistence and toxicity of fluorinated HAAs [16,22,23], there is a need for further investigation of their chlorinated and brominated counterparts which are commonly detected in aquatic ecosystems.

The ubiquity of HAAs has prompted several traditional endpoint toxicity studies using a range of model aquatic organisms [16,22,24,25,26,27,28]. Under laboratory conditions, exposure to DCAA and TCAA was found to exhibit phytotoxicity for two algal species (*Myriophyllum spp.* and *Lemna gibba*) where the wet mass and root length of the aquatic plants was inhibited by the presence of HAAs [16,22,25]. Acute dibromoacetic acid (DBAA) exposure to Japanese medaka (*Oryzias latipes*) negatively impacted fish length and weight [26]. DCAA has been found to hinder reproduction in zebrafish (*Danio rerio*) embryos [27,28]. Embryos exposed to varying concentrations of DCAA resulted in concentration-dependent fluxes in heartrate and blood flow as well as malformations in the mouth and notochord stemming from oxidative stress caused by contaminant exposure [27,28]. The LC_50_ and perturbations to reproduction with DBAA in the water flea (*Daphnia magna*), sheepshead minnow (*Cyprinodon variegatus*) and algae (*Ischryis galbana*) [29] showed that DBAA exposure negatively impacted the growth rates of *I. galbana* with higher exposure concentrations (>97.8 mg/L) [29]. The chronic inhibitory concentration (IC_25_, 284.6 mg/L) was comparable to the 96-hour LC_50_ (259.9 mg/L) for DBAA exposure in *D. magna* [29]. Interestingly, LC_50_ values for *D. magna* were similar for both acute (96 h) and chronic (21 day) experiments [29] suggesting that chronic toxicity can be predicted from acute exposure, which is further supported by the low calculated acute to chronic ratios. These findings determined that after 72 h acute tests, elevated concentrations of DCAA and TCAA led to growth inhibition of *I. galbana* at concentrations of 104.5 and 447.0 mg/L, respectively [29]. In *C. variegatus* the acute LC_50_ of DCAA was determined to be 321.6 mg/L, which was the same LC_50_ observed after 24, 48, 72 and 96 h experiments [29]. Chronic TCAA exposure studies demonstrated in both *D. magna* and *C. variegatus* that survival was as sensitive as reproductive tests when assessing conventional toxicity [29]. For all HAAs (DBAA, DCAA and TCAA) studied, a strong relationship between the concentration of the compound, the health of the organisms tested, and the pH of the media was observed [29]. Additionally, in chronic multigenerational studies with *D. magna*, exposure to HAAs decreased the rate of sexual maturity and the number of offspring produced during reproduction, which were more apparent in subsequent generations [21]. These studies using traditional toxicity endpoints demonstrate that HAAs can adversely impact many aquatic organisms and may have far-reaching ecological impacts.

Previous studies have also investigated the toxic mode of action of HAAs to various organisms [23,24,30,31]. Exposure to DCAA and DBAA induced deoxyribonucleic acid (DNA) damage and mutagenic activity in the bacteria *Escherichia coli* and *Salmonella typhimurium* [30]. In mammalian murine models, exposure to DCAA and DBAA resulted in developmental toxicity in rat embryos, leading to malformed neural tubes [32]. Studies have collectively determined that exposure to HAAs, notably DCAA and TCAA, have led to teratogenic malformations in offspring in neural and cardiac systems [33,34,35]. Following exposure to trichloroethylene, metabolites DCAA and TCAA were found to contribute to liver cancer in mice when administered orally through contaminated drinking water [36]. It has also been suggested that brominated DBPs pose a larger cytotoxic and genotoxic risk than their chlorinated counterparts [37,38], but this may be species-dependent. Another murine-based study determined the inhibitory role on the enzyme pyruvate dehydrogenase kinase onset by exposure to DCAA [39]. This enzymatic complex is responsible for the reactions which convert pyruvate into acetyl coenzyme A (acetyl-CoA) [40]. Rainbow trout (*Oncorhynchus mykiss*) also exhibited elevated blood lactate concentrations stemming from the inhibition of pyruvate dehydrogenase kinase [24], as was also observed in a murine study [39]. In the Japanese medaka (*O. latipes*), exposure to DCAA increased concentrations of glycogen, which is likely indicative of disruptions in the enzymatic pathways involved in glycolysis [41]. Lastly, acute toxicity tests using trifluoroacetate (TFAA) on multiple algal strains concluded that the toxic mode of action of HAAs may stem from the inhibition of the citric acid (TCA) cycle, as the algal growth began to recover with additions of citric acid to the media [23].

Although these aforementioned studies have outlined the toxicological impacts of HAAs on aquatic organisms, no study has investigated, to date, the toxic mode of action in the model organism, *D. magna*, at the molecular level. *D. magna* is a commonly studied water flea due to its ecological relevance and prominent use in ecotoxicity as a model organism for aquatic environments due to their heightened sensitivity to anthropogenic contaminants [42,43]. For these reasons, *D. magna* has been used in environmental metabolomic studies to investigate the mode of action of a range of environmentally persistent organic pollutants and metals [44,45,46,47]. These studies have collectively identified the toxic mode of action with sub-lethal exposure, which is more relevant given the environmental concentrations of many pollutants in aquatic ecosystems. Previous studies with *D. magna* have shown that metabolomic approaches are able to detect short-term perturbations to the metabolite profile and that it is a promising approach to elucidate the impacts of pollutants on aquatic organisms [48,49,50]. Here liquid chromatography with tandem mass spectrometry (LC-MS/MS) was used to decode the metabolic responses of *D. magna* to three commonly observed HAAs (DCAA, TCAA and DBAA) in acute sub-lethal exposure studies. This method targets amino acids and derivatives, neurotransmitters, nucleosides/nucleotides, saccharide derivatives, vitamins, polyamines and carboxylic acids and has been used to assess sub-lethal toxicity in previous studies [51,52,53,54]. Previous studies reported that exposure to HAAs leads to disruptions in energy metabolism [23,24,39,41], and, as such, it is hypothesized that *D. magna* will also exhibit energy disruption with contaminant exposure. Therefore, the objective of this study is to determine how the polar metabolic profile of *D. magna* is altered with acute sub-lethal exposure to DCAA, TCAA and DBAA. Several different concentrations of DCAA, TCAA and DBAA were also used to test how metabolic perturbations and the toxic mode of action are potentially correlated to HAA exposure concentration. To the best of our knowledge, this is the first study that uses a targeted metabolomic approach to investigate the impacts of sub-lethal HAA exposure to *D. magna*.

## 2. Results

### 2.1. Multivariate Analysis of Metabolic Changes with HAA Exposure

**Figure 1:**
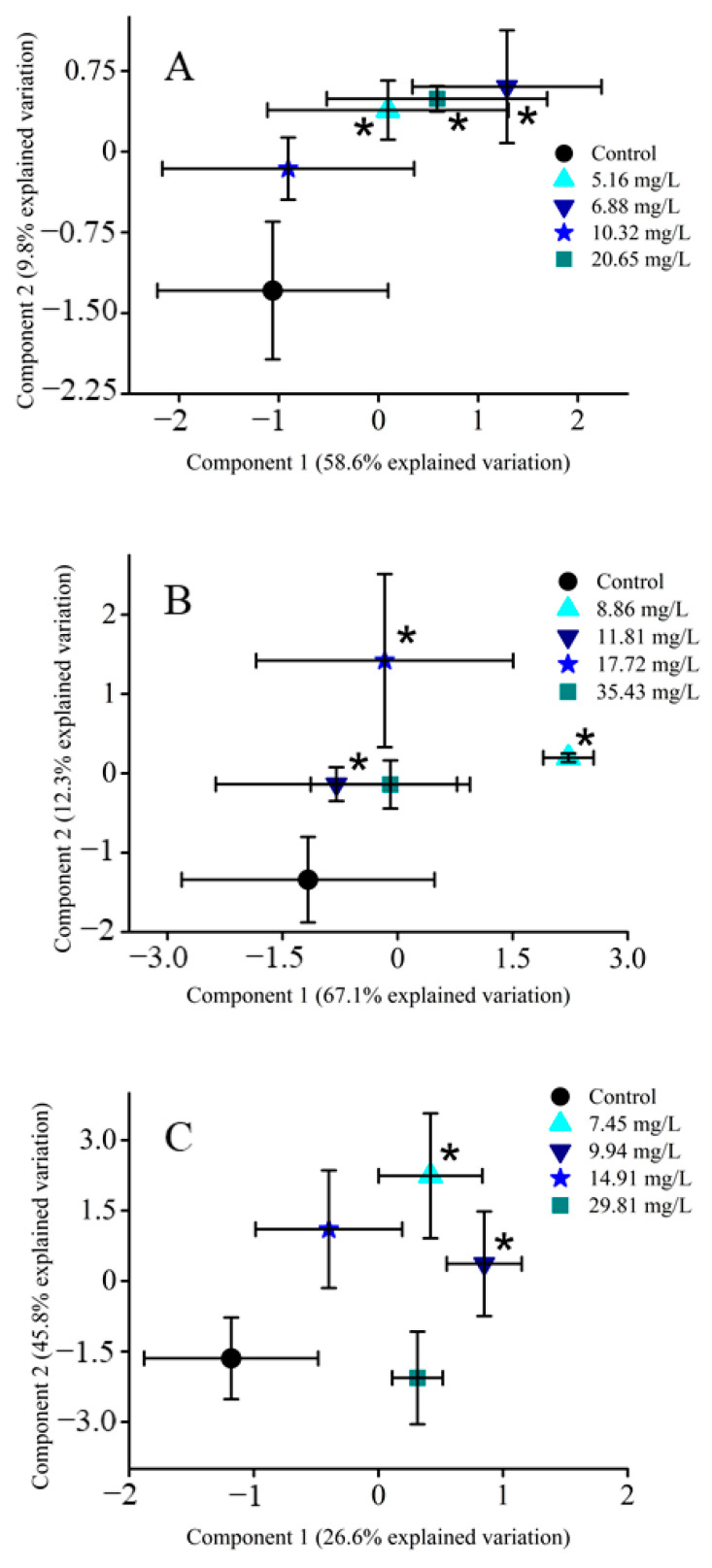
Partial least squares-discriminant analysis (PLS-DA) scores plots of averaged (*n* = 12) metabolite data sets from all treatment groups. Statistical significance (*p* < 0.05) is outlined by the presence of an asterisk (*). (**A**) PLS-DA of DCAA exposure, (**B**) PLS-DA of TCAA exposure, (**C**) PLS-DA of DBAA exposure.

Principal component analysis (PCA) and partial least squares—discriminant analysis (PLS-DA) are commonly used to screen for overall changes in measured metabolites relative to the control (unexposed organisms) [55,56]. With exposure to all three HAAs (DCAA, TCAA and DBAA), shifts in the *D. magna* metabolome were observed (Figure 1 and Supplementary Materials Figure S1), but not all changes were statistically significant. In addition, separation was more significant with PLS-DA than with PCA, which is consistent with the supervised (PLS-DA) and unsupervised (PCA) nature of these multivariate analysis methods [57]. For all three HAAs studied, components 1 and 2 for both PCA and PLS-DA represented more than 68% of the total variation (Figure 1 and Supplementary Materials Figure S1). Components 3 and 4 were also examined, but these explained less of the overall differences relative to the unexposed groups (<8.5%; Supplementary Materials Figure S1). As such, comparisons between the different HAAs focused on components 1 and 2, as these were representative of most of the variation for both PCA and PLS-DA.

After sub-lethal exposure, both PCA and PLS-DA exhibited a shift in the overall metabolic profile relative to the control. The DCAA-exposed groups were separated from the control, indicating that the metabolic profile was altered with exposure (Figure 1A and Supplementary Materials Figure S1A–C). Interestingly, PLS-DA identified a statistically significant (*p* < 0.05) separation relative to the control with TCAA exposure (Figure 1B and Supplementary Materials Figure S1F), whereas PCA did not show the same distinction between exposed and control groups (Supplementary Materials Figure S1D,E). With DBAA exposure, the *D. magna* metabolic profile was distinct from the control in both PCA and PLS-DA (Figure 1C and Supplementary Materials Figure S1G–I). Notably, the highest exposure concentration (29.81 mg/L) was significantly different after PCA (Supplementary Materials Figure S1G), suggesting that this concentration altered the profile the most. PLS-DA identified significant (*p* < 0.05) shifts with the two lower concentrations (7.45 and 9.94 mg/L) of DBAA (Figure 1C). Overall, each of the HAAs exhibited different clustering patterns, but in general induced a change in the metabolic profile that was identified by both PCA and PLS-DA. The overall clustering in both PCA and PLS-DA, except for DBAA, did not appear to follow any concentration-dependence.

### 2.2. Changes in Specific Metabolites with HAA Exposure

#### 2.2.1. Dichloroacetic Acid (DCAA) Metabolite Changes

**Figure 2:**
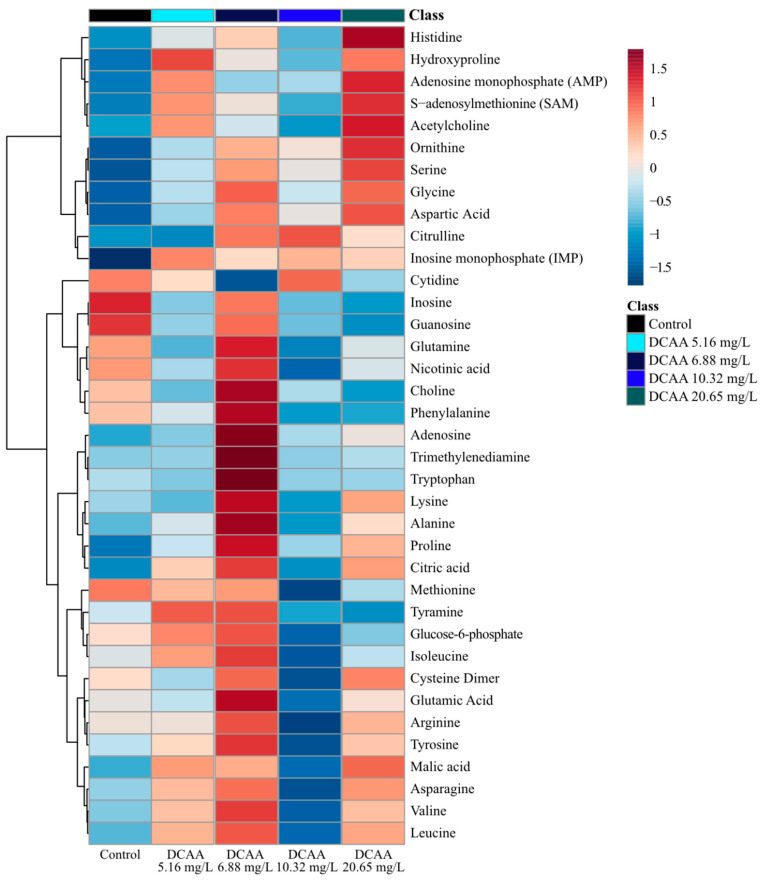
Analysis of averaged metabolite concentrations (*n* = 12) where 0 is the average metabolite concentrations across all treatment groups (dichloroacetic acid (DCAA) exposure and the control). The color gradient (blue to red) shows the relative difference compared to the average metabolite concentration, and metabolites are linked on the left y-axis. The heatmap arranged by dendrogram on the x-axis is found in the Supplementary Materials (Figure S3).

Sub-lethal exposure to DCAA altered the concentration of several metabolites in *D. magna* (Figure 2). A heatmap clusters and organizes metabolite changes across two axes: the x-axis categorizes the exposure and control groups based on similarities in the relative metabolic profile; the y-axis separates the quantified metabolites by similarities in the response of the metabolite concentrations. The changes presented in the heatmap display shifts in specific metabolites relative to the average concentration of each metabolite measured. Consistent with the PCA and PLS-DA analyses, no apparent trends with respect to changes in metabolites with exposure concentration were observed based on the clustering and hierarchy (Figure 2, Supplementary Materials Figure S3). The second exposure group (6.88 mg/L) increased the concentration of metabolites, except for cytidine, adenosine monophosphate (AMP) and acetylcholine (Figure 2 and Supplementary Materials Figure S4). In the third exposure group (10.32 mg/L) there was a decrease in the metabolite concentrations of *D. magna*, and this response was the most similar to that of the unexposed control group (Figure 2, Supplementary Materials Figure S3). Interestingly, the lowest and highest exposure groups (5.16 and 20.65 mg/L of DCAA) resulted in the most significant distinction relative to the control, which is consistent with multivariate statistics performed such as PCA and PLS-DA (Figure 2, Supplementary Materials Figure S3). The two exposure groups (5.16 and 20.65 mg/L) resulted in the opposite response (increase versus decrease) of metabolites when compared to the control. These observed changes suggest that DCAA exposure may alter metabolites non-monotonically in *D. magna*.

**Figure 3:**
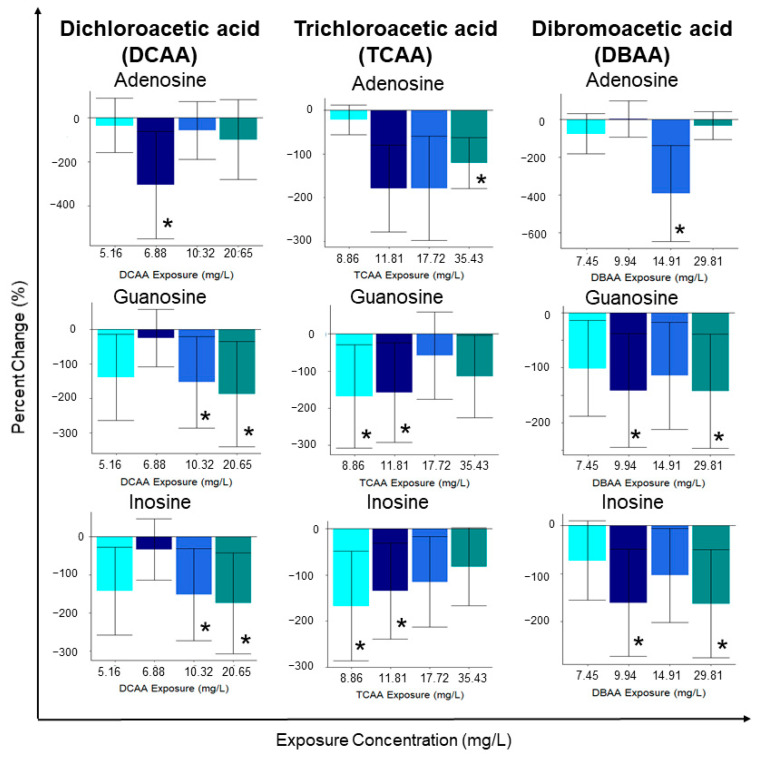
Averaged (*n* = 12) individual metabolite percent changes following sub-lethal exposure to varying concentrations of DCAA exposure (5.16, 6.88, 10.32 and 20.65 mg/L), trichloroacetic acid (TCAA) exposure (8.86, 11.81, 17.72 and 35.43 mg/L) and dibromoacetic acid (DBAA) exposure (7.45, 9.94, 14.91 and 29.81 mg/L). Statistical significance (*p* < 0.05) was determined using a two-tailed, equal variance *t*-test and is outlined with the presence of an asterisk (*).

Relative fold change analysis of metabolite concentrations showed that the most significantly (*p* < 0.05) altered metabolites included: adenosine, guanosine and inosine, all of decreased with all contaminant exposures (Figure 3). The concentration of guanosine and inosine significantly (*p* < 0.05) decreased following exposure to the two highest concentrations (10.32 and 20.65 mg/L of DCAA) of the contaminant (Figure 3). In addition to the significantly altered metabolites, the concentration of most of the quantified metabolites was downregulated compared to the unexposed control group (Supplementary Materials Figure S4). Interestingly, in addition to the significant decreases in nucleosides adenosine, guanosine and inosine, other nucleosides and related metabolites (adenosine monophosphate (AMP), inosine monophosphate (IMP) and uridine) also decreased. Exposure to DCAA decreased the concentrations of AMP, IMP and uridine in *D. magna* (Supplementary Materials Figure S4). The concentration of several amino acids, including serine, glycine, aspartic acid, proline and methionine, were downregulated with all exposure concentrations of DCAA (Supplementary Materials Figure S4). Other metabolites which play a role in the citrate cycle, including malic acid and citrate, were also downregulated with DCAA exposure (Supplementary Materials Figure S3). A strong positive correlation was observed between the concentration of inosine and guanosine, suggesting that the mode of action of the contaminant may impact both nucleosides during its onset (Supplementary Materials Figure S5). Lastly, in agreement with the observed trends in select amino acids, a Pearson correlation analysis resulted in high correlation coefficients between the metabolites serine, glycine and proline (Supplementary Materials Figure S5). Fluxes in isoleucine, leucine and valine were also strongly correlated with serine, glycine and proline, which suggests ties to metabolic pathways linked to amino acid biosynthesis/degradation (Supplementary Materials Figure S5).

#### 2.2.2. Trichloroacetic Acid (TCAA) Metabolite Changes

**Figure 4:**
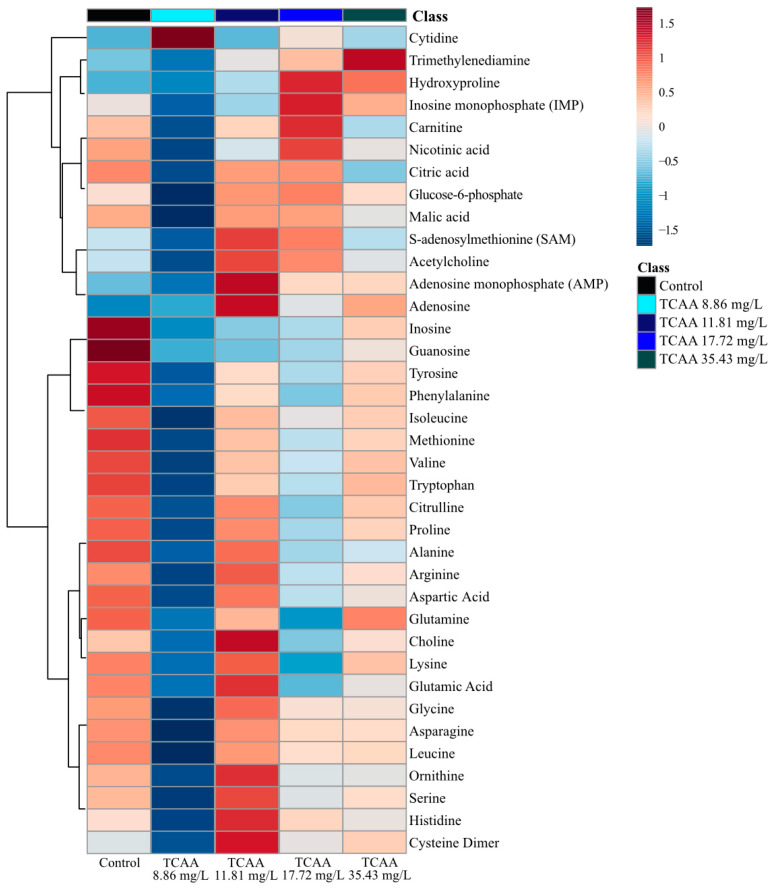
Analysis of averaged metabolite concentrations (*n* = 12) where 0 is the average metabolite concentrations across all treatment groups (TCAA exposure and the control). The color gradient (blue to red) shows the relative difference compared to the average metabolite concentration, and metabolites are linked on the left y-axis. The heatmap arranged by dendrogram on the x-axis is found in the Supplementary Materials (Figure S7).

The analysis of metabolites after TCAA exposure did not reveal any concentration-dependence (Supplementary Materials Figure S8) as observed in PCA and PLS-DA (Figure 1). Exposure to the second and third exposure concentrations (11.81 and 17.72 mg/L of TCAA) resulted in metabolic perturbations from the unexposed control group (Figure 4, Supplementary Materials Figure S7). The lowest and highest concentrations (8.66 and 35.43 mg/L of TCAA) were identified as having the most significant distinction from the unexposed group (Figure 4, Supplementary Materials Figure S7). Overall, the lowest TCAA exposure concentration (8.86 mg/L) decreased the concentration of all metabolites except cytidine (Figure 4). The highest exposure concentration (35.43 mg/L of TCAA) led to increased trimethylenediamine (Figure 4, Supplementary Materials Figure S7) relative to the control.

Individual metabolite changes also provide evidence that sub-lethal exposure to TCAA led to metabolic perturbations in *D. magna*, but non-monotonically (Supplementary Materials Figure S8). TCAA exposure resulted in significantly (*p* < 0.05) downregulated concentrations of adenosine, guanosine and inosine (Figure 3). In addition, cytidine and citric acid were downregulated relative to the unexposed control group (Supplementary Materials Figure S8). Sub-lethal exposure also downregulated select amino acids relative to the control; this includes decreases in glutamine, alanine, proline, valine, leucine, isoleucine, tyrosine and phenylalanine at all TCAA exposure concentrations (Supplementary Materials Figure S8). It is important to note that TCAA did not decrease all nucleoside metabolites as observed with DCAA. Metabolites AMP, IMP and uridine both up and downregulated with varying exposure concentrations (Supplementary Materials Figure S8). The metabolite percent changes further support that the mode of action of TCAA varies with exposure concentration. A Pearson correlation analysis exhibited a high correlation between neurotransmitters and nucleosides including nicotinic acid, carnitine and IMP (Supplementary Materials Figure S9). TCAA exposure resulted in a strong positive correlation between the concentrations of several amino acids (Supplementary Materials, Figure S9). These include asparagine, leucine, alanine, proline, glutamate, valine and isoleucine, all of which were downregulated after sub-lethal exposure to TCAA (Supplementary Materials Figures S8 and S9). Interestingly, the Pearson correlation analysis did not provide any evidence of existing correlations between nucleosides adenosine, inosine and guanosine, as observed with exposure to DCAA and DBAA (Supplementary Materials Figure S9).

#### 2.2.3. Dibromoacetic Acid (DBAA) Metabolite Changes

**Figure 5:**
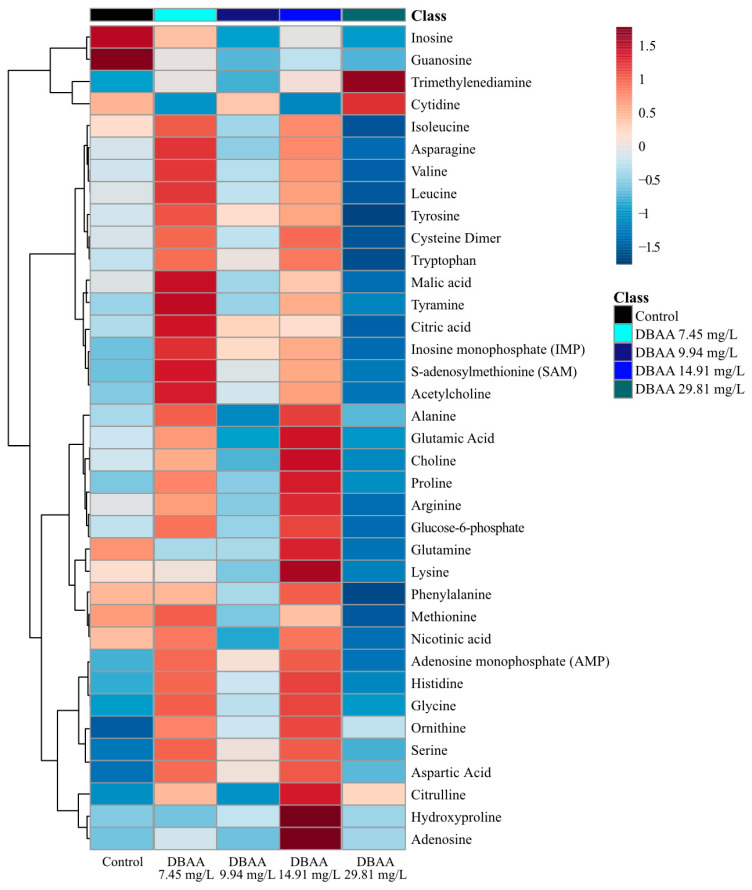
Analysis of averaged metabolite concentrations (*n* = 12) where 0 is the average metabolite concentrations across all treatment groups (DBAA exposure and the control). The color gradient (blue to red) shows the relative difference compared to the average metabolite concentration, and metabolites are linked on the left y-axis. The heatmap arranged by dendrogram on the x-axis is found in the Supplementary Materials (Figure S11).

Consistent with the PCA and PLS-DA analyses, the changes in the analyzed metabolites did not follow clustering and hierarchy in a concentration-dependent manner with exposure (Figure 5, Supplementary Materials Figure S11). Based on the average concentration of all metabolites, there are two distinct responses to sub-lethal DBAA exposure in *D. magna* (Figure 5, Supplementary Materials Figure S11). The hierarchy and clustering shows that the response from the lowest and second highest exposure concentrations (7.45 and 14.91 mg/L of DBAA) were the most distinct from that of the unexposed control group (Figure 5, Supplementary Materials Figure S11). In these exposure groups (7.45 and 14.91 mg/L) metabolite concentrations increased relative to the total average (Figure 5, Supplementary Materials Figure S11). The concentration of the metabolites in the control group decreased when compared to the average, with exceptions for inosine, guanosine, glutamine, methionine, nicotinic acid and phenylalanine (Figure 5). Furthermore, the second lowest and highest exposure concentrations (9.94 and 29.81 mg/L of DBAA) decreased across the concentrations of metabolites in *D. magna* (Figure 5, Supplementary Materials Figure S11). The second exposure concentration (9.94 mg/L of DBAA) resulted in lower concentrations of some metabolites, namely in inosine, guanosine, glutamine, lysine, methionine, nicotinic acid and phenylalanine (Figure 5, Supplementary Materials Figure S11). The highest exposure group (29.81 mg/L) decreased the concentration of metabolites, except for trimethylenediamine, cytidine and citrulline.

Sub-lethal exposure to DBAA led to a statistically significant (*p* < 0.05) downregulation in the concentrations of adenosine, guanosine and inosine, except for the lowest exposure concentration (Figure 3; Supplementary Materials Figure S12). Both inosine and guanosine were significantly (*p* < 0.05) downregulated at the highest and second highest exposure concentrations of DBAA (29.81 and 9.94 mg/L; Figure 3). As observed with DCAA and TCAA, DBAA led to decreased nucleoside concentrations, suggesting a common mode of action in the contaminant class which is independent on the type of halogen in the HAA. DBAA exposure decreased amino acid and nucleoside concentrations with all exposure concentrations, which is consistent with observations from DCAA and TCAA exposure. DBAA exposure resulted in the downregulation of serine, glycine, threonine and aspartate (Supplementary Materials Figure S12). In addition to significant (*p* < 0.05) decreases in the nucleosides adenosine, inosine and guanosine, other nucleosides—AMP, IMP and uridine—were also downregulated (Supplementary Materials Figure S12). For many metabolites, the response of the highest concentration exposure (DBAA 29.81 mg/L) was unique compared to other DBAA exposure groups. Specifically, histamine, histidine, S-adenosylmethionine (SAM), proline, dopamine, acetylcholine, citric acid and cytidine all increased with the highest DBAA exposure concentration. As with DCAA and TCAA, DBAA exposure also produced a strong positive correlation between the concentrations of the amino acid phenylalanine, isoleucine, leucine, valine and asparagine (Supplementary Materials Figure S13). The correlation analysis supports the link between metabolite concentrations of inosine and guanosine, which suggests that these are linked in the toxic mode of action of DBAA (Supplementary Materials Figure S13). Interestingly, the Pearson correlation analysis does not indicate a strong positive correlation between the nucleoside adenosine to inosine and guanosine (Supplementary Materials Figure S13).

### 2.3. Biochemical Pathway Analysis

Using MetaboAnalyst (version 4.0), biochemical perturbations in the metabolome of *D. magna* were assessed using pathway analysis [58]. Following the sub-lethal exposure to DCAA, TCAA and DBAA, no significant (*p* < 0.05) disruptions in the metabolome of the exposed organisms were detected. Pathway analysis using a 90% confidence interval (*p* < 0.10) threshold identified the disruption of several biochemical pathways in *D. magna* (Table 1). Sub-lethal exposure to the highest experimental concentration of DCAA (20.65 mg/L) resulted in notable perturbations (*p* < 0.10) in the purine metabolism pathway and in the TCA cycle (Table 1 and Supplementary Materials Figure S14). *D. magna* exposure to concentrations of TCAA led to changes in many pathways (Table 1; Supplementary Materials Figure S15). Of the disturbed pathways highlighted, the TCA cycle was impacted by TCAA-exposed *D. magna* and was also observed following exposure to DCAA and DBAA (Table 1). Exposure to TCAA additionally led to disruptions in pathways relating to amino acid metabolism, biosynthesis and degradation of phenylalanine, tyrosine and tryptophan, valine, leucine, isoleucine, cysteine and methionine (Table 1). The sub-lethal exposure of *D. magna* to DBAA led to disruptions in the thiamine and tyrosine metabolism pathways, the TCA cycle and purine metabolism pathways (Table 1 and Supplementary Materials Figures S16 and S17).

## 3. Discussion

Metabolic profiles for all three HAAs (DCAA, TCAA and DBAA) varied the concentrations of several metabolites, including adenosine, inosine and guanosine, after acute sub-lethal exposure (Figure 3). Both supervised and non-supervised multivariate statistics (Figure 1 and Supplementary Materials Figure S1) did not show any clear trends between separation clusters and exposure concentration for all the three HAAs studied. This may be due to biological variability in *D. magna*, which may limit the statistical significance of results as well as different responses of specific metabolites with each HAA concentration. The latter also suggests a non-monotonic toxic mode of action of sub-lethal HAA exposure. Several other studies have observed that organic pollutant exposure can yield a varied metabolite profile change in *D. magna* that is non-monotonic [59,60,61,62]. These studies have also found that metabolite changes in *D. magna* are unique to the exposure concentration in acute studies [59,60,61,62] (Supplementary Materials Figures S4, S8, and S12). In this study, the analysis of metabolite clusters (Figure 2, Figure 4 and Figure 5 and Supplementary Materials Figures S3, S7 and S11) also support that the different HAAs invoke varied responses to *D. magna* metabolic processes when exposed to a range of sub-lethal concentrations. This is further supported by the relative change of individual metabolites (Supplementary Materials Figures S4, S8 and S12), which shows that HAAs result in a non-monotonic response to *D. magna*. However, exposure to all three HAAs (DCAA, TCAA and DBAA) led to significant (*p* < 0.05) decreases in the concentration of the same nucleosides, including adenosine, guanosine and inosine, which suggests a similar generic response to HAAs. As such, these metabolites may be suitable bioindicators for HAA exposure monitoring in *D. magna*.

In addition to detecting variations in the metabolic profile, the analysis of how these specific metabolites disrupt metabolic pathways is necessary and informative with respect to understanding the toxic mode of action. Nucleosides and nucleotides such as adenosine and guanosine play key roles in the mitochondria of cells and phosphorylation mechanisms [63]. Nucleosides and nucleotides are important for the physiology of organisms. In *D. magna*, guanosine and its phosphorylated derivatives are present in the embryos to provide essential building blocks for the growth of the neonates [64,65]. Adenine, guanine and hypoxanthine are nucleobases which are precursors in the synthesis of the purine nucleosides, adenosine, inosine and guanosine through the addition of ribose [66]. The addition of ribose and phosphate groups to the nucleobases generates nucleotides which can be used in the synthesis of ribonucleic acid (RNA) [67]. Comparatively, the addition of deoxyribose leads to precursors which are used by the organism in vivo to synthesize DNA [68]. Virtually all biological processes rely on the presence of proteins, RNA and DNA in order to control cells and organisms [69]. Nucleosides and nucleotides are synthesized and degraded through purine metabolism pathways [66]. Purine pathways have been described as a biochemical mechanism used by organisms to meet energetic and molecular demands [66]. Exposure to DCAA and DBAA in *E. coli* and *S. typhimurium* resulted in mutagenic activity and DNA damage in the organisms [30]. The significant downregulation in adenosine, guanosine and inosine with exposure to HAAs implies a disruption in the ability of *D. magna* to regulate the purine metabolism pathway. This finding is further confirmed by the MetaboAnalyst pathway analysis results (Table 1) for all three HAAs (DCAA, TCAA and DBAA), which indicated disruptions in the purine metabolism pathway. Interestingly, the disruption in the purine metabolism pathway was more apparent in the dihalogenated contaminants (DCAA and DBAA). Toxicity studies using TFAA indicated that its metabolized intermediate, monofluoroacetate, leads to metabolic disruption following defluorination reactions in the organism [22,23]. The increased toxicity of dihalogenated HAAs (DCAA and DBAA) stems from the contaminants having a more comparable chemical structure to acetyl needed in acetyl-CoA production, which is then introduced into the TCA cycle compared to its trihalogenated counterparts (TCAA) [23,70].

The purine pathway is also impacted by HAA exposure and corresponds to rate limiting steps involving other metabolites quantified in *D. magna*. Glutamic acid, glutamine, glycine, glucose-6-phosphate, histidine, IMP and aspartic acid are cofactors and intermediates within the purine metabolism pathway [66]. These metabolites were downregulated in *D. magna* following DCAA, TCAA and DBAA exposure (Supplementary Materials Figures S4, S8 and S12). The diverse response of metabolites after exposure to different concentrations of these contaminants further supports the non-monotonic mode of action onset by the presence of HAAs. The non-monotonic response in *D. magna* may also suggest rate-limiting steps in the dehalogenation reactions required to induce toxicity [23]. The purine metabolism pathway contains intermediates which are linked to the TCA cycle, including pyruvate, a terminal product from glycolysis and the synthesis of fumarate [66]. Interestingly, pathway analysis determined that the sub-lethal exposure of *D. magna* to DCAA, TCAA and DBAA led to notable disruptions in the TCA cycle (Table 1). The non-monotonic response in TCA cycle intermediates, citric acid and malic acid supports the findings previously presented (Supplementary Materials Figures S4, S8 and S12). In studies using TFAA, the authors determined that the inhibition of the TCA cycle stems from the inhibition of the aconitase enzyme present in *D. magna* [22,23,71]. The aconitase enzyme plays a central role in the TCA cycle, as it is responsible for the isomerization of tricarboxylic acids in the TCA cycle [72,73]. Aconitase catalyzes the conversion of citrate to isocitrate in the first stages of the metabolic pathway [73]. Inhibition of the TCA cycle would lead to interferences with the generation of ATP and other known energy-related metabolites [22,72]. Toxicity studies using other halogenated acetates/acetic acids including monofluoroacetate and TFAA have reported disruptions within energy metabolism due to the inhibition of the corresponding enzymes [23,74]. Consequently, similarities in the disrupted pathways following DCAA, TCAA and DBAA exposure imply a class-based response which is independent of the types or number of halogens on the HAAs.

The exposure of *D. magna* to each of the HAAs also disrupted the thiamine metabolism and tyrosine metabolism pathways (Table 1). Thiamine and thiamine phosphate esters are crucial cofactors in integral biochemical pathways such as glycolysis, purine metabolism and the TCA cycle [75]. In organisms, thiamine acts as a cofactor in biotic and abiotic stress mechanisms [75]. Following the sub-lethal exposure to DCAA, the concentration of thiamine remained comparable to what was measured in the control organisms (Supplementary Materials, Figure S4). However, exposure to TCAA and DBAA led to decreases in thiamine relative to the unexposed group (Supplementary Materials Figures S8 and S12). It has been reported that fluxes in the purine metabolism and histidine biosynthesis pathways can influence the de novo biosynthesis of thiamine and its phosphate esters [76]. The purine intermediate, aminoimidazole carboxamide, inhibits thiamine synthesis [76]. The degree of the disruption in interconnected pathways including the TCA cycle and purine metabolism pathways may also explain the varied response of thiamine in DCAA when compared to the metabolite response in TCAA and DBAA. Thiamine metabolism may be disrupted under conditions of stress both in plants [77] and in humans [78]. *D. magna* exposure to HAAs incited a disruption to the thiamine metabolism likely as a result of downregulations in the purine metabolism pathway. Included as intermediates in the thiamine metabolism pathway are metabolites which are important in other biochemical processes including glycine and pyruvate. Lastly, the metabolite tyrosine also plays a role in the thiamine metabolism pathway [75]. The concentration of tyrosine following DCAA and DBAA exposure did not change relative to the control group (Supplementary Materials Figures S4 and S12). However, following TCAA exposure, the concentration of tyrosine in all experimental groups was downregulated (Supplementary Materials Figure S8). The response of tyrosine did not follow an exposure concentration-dependence and further supports that HAA exposure leads to a non-monotonic response in *D. magna*. Tyrosine metabolism pathways can also impact phenylalanine metabolism pathways [79]. Both the phenylalanine and tyrosine metabolic pathways are important for the production of fumarate, which is an intermediate in the TCA cycle [79,80].

## 4. Materials and Methods

### 4.1. Culturing of Daphnia Magna

*Daphnia magna* have been consistently cultured since 2013 with the original culture purchased from Ward Science Canada (Rochester, NY, USA). The *Daphnia* were cultured based on guidelines from the Ontario Ministry of the Environment [81]. The culture is maintained at a temperature of 22 °C with a 16 h: 8 h light: dark cycle. Municipal tap water which has been dechlorinated using 0.1 M sodium thiosulfate (Hach Company, Loveland, CO, USA) and aerated for a minimum of 48 h before use was used as culturing medium. The water has a hardness of approximately 120 mg/L of CaCO_3_ (this is consistent with the regional freshwater conditions). Twice a week, the daphnids were fed with algae, and approximately 40% of the old water was changed and replaced with fresh dechlorinated water. *D. magna* were fed with a 1:1 mixture of *Raphidocelis* (*Pseudokirchneriella*) *subcapitata* and *Chlorella vulgaris* grown in Bristol media [82]. In addition to algae, 1 µg/L of each selenium and cobalamin were added to the culture media to ensure that organism health and reproduction reached the standards set by the Ontario Ministry of the Environment [81].

### 4.2. Lethal Concentration (LC_50_) Determination of Select Halogenated Acetic Acids (HAAs)

DCAA (>99% purity) and DBAA (97% purity) were purchased from Sigma Aldrich (Mississauga, ON, Canada). TCAA (>99% purity) was purchased from Fisher Scientific (Toronto, ON, Canada). To obtain the 48-h LC_50_ for the HAAs, a lethal toxicity test was conducted using DCAA, TCAA and DBAA, along with NaCl (Amresco, Ohio; 99.9%) as a reference toxicant. The LC_50_ test was performed following the guidelines of the Environment Canada Biological Test Method for *Daphnia magna* [83]. Neonates were exposed to DCAA, TCAA or DBAA in concentrations ranging from 62.25 mg/L to 2000 mg/L (0, 62.25, 125, 250, 500, 1000 and 2000 mg/L), and were divided into three replicates per concentration (*n* = 10) and kept at a density lower than the recommended 1 daphnid per 15 mL of media [83]. A secondary group of neonates were exposed to the reference toxicant, NaCl (10 mg/L–10000 mg/L), with triplicates of 10 neonates used for each given concentration (0, 10, 100, 1000, 5000 and 10,000 mg/L). At the end of the 48-h exposure period, the daphnids were microscopically observed to determine the number of deceased organisms, and death was defined by a lack of cardiac movement. The results were calculated using the Logit function, where the LC_50_ is calculated based on the number of neonates deceased per given exposure using a corrected proportion to account for natural deaths within the population [84].

(1) $$Logit=\ln\left(\frac{Corrected\;Proportion}{1-Proportion}\right),$$

(2) $$Corrected\;Proportion=\;\frac{Proportion-\;Proportion_{0}}{1-Proportion_{0}},$$

(3) $$Proportion=\;\frac{Dead\;neonates}{Total\;neonates}.$$

where Proportion is equal to the proportion of the control groups. The resulting data were plotted by log concentration against the logit to obtain a scatter graph. The intercept is the calculated LC_50_ for the experiment. After a 48-h acute exposure to the test contaminants, the LC_50_ for the respective contaminants was calculated (Table 2).

The concentration of the HAAs, including DCAA, TCAA and DBAA, was confirmed using the method of Xie et al. [85] using gas chromatography-mass spectrometry (GC-MS) and is detailed in the Supplementary Materials (Supplementary Materials Tables S1 and S2). The concentration of the reference toxicant was quantified using ion chromatography using the relative concentration of the chloride anion as a marker for the concentration of NaCl (Supplementary Materials Section 2).

### 4.3. Sub-Lethal Exposure of DCAA, TCAA and DBAA to D. magna

**Figure 6:**
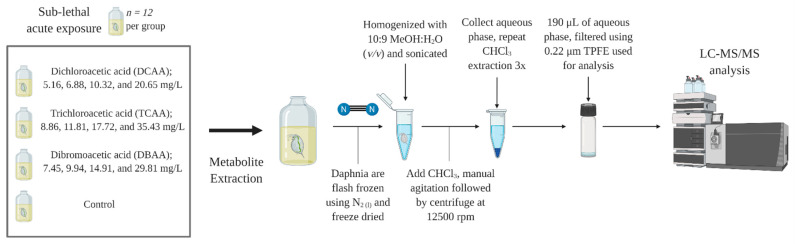
Experimental workflow of acute sub-lethal exposure of halogenated acetic acids (dichloroacetic acid; DCAA, trichloroacetic acid; TCAA, and dibromoacetic acid; DBAA) and subsequent extraction of polar metabolites from *Daphnia magna*. Figure created with BioRender.com.

DCAA, TCAA and DBAA were used in 48-h sub-lethal exposure experiments with *D. magna*. A visual representation of the workflow used in this study is found in Figure 6. Concentrations below the respective LC_50_ values (Table 1) were chosen, and the *Daphnia* were exposed to the compounds. Adult daphnids were exposed to either DCAA (5.16, 6.88, 10.32, and 20.65 mg/L), TCAA (8.86, 11.81, 17.72, and 35.43 mg/L) or DBAA (7.45, 9.94, 14.91, and 29.81 mg/L). These concentrations represent 1/40th, 1/30th, 1/20th and 1/10th of the respective experimentally determined LC_50_ values (Table 2). For each treatment group, including the controls, the *Daphnia* (*n* = 12) were kept in 20 mL scintillation vials maintaining the loading density of 1 daphnid per 15 mL of exposure media (Figure 6). Due to the limit of detection/quantification requirements not met by the GC-MS detection method used for LC_50_ concentrations, the nominal concentrations of the HAAs were confirmed using liquid chromatography tandem mass spectrometry [86,87] (LC-MS/MS; Supplementary Materials Tables S3–S7). The culturing conditions described in Section 2.1 were maintained during the exposure, and *Daphnia* were fed after 24 h with *R. subcapitata* 0.1 mg/L of algae per daphnid. At the end of the exposure, the *Daphnia* were removed from the water and rinsed with clean dechlorinated and aerated water. *Daphnia* were then flash-frozen using liquid nitrogen to halt enzymatic activity (Figure 6). The daphnids were then lyophilized for 24 h (ModulyoD, ThermoFisher, Toronto, ON, Canada, Figure 6). The samples were kept frozen at −25 °C until extraction, and their metabolite profile was analyzed using LC-MS/MS.

### 4.4. Metabolite Extraction and LC-MS/MS Analysis

*Daphnia* metabolites were extracted based on the method of Jeong and Simpson [53]. This method was specifically developed to isolate polar metabolites from individual daphnids and has high recovery rates for the suite of metabolites analyzed [53]. Briefly, the *Daphnia* were individually placed into 2.5 mL Eppendorf tubes (Eppendorf, Mississauga, ON, Canada) and 160 µL of 10:9 methanol (Fisher Scientific, Toronto, ON, Canada; 99.9% purity): water (*v*/*v*) was added to each tube (Figure 6). The extraction was performed over ice to preserve the stability of the metabolites during the procedure. The samples were manually ground with a motorized pestle to homogenize the tissue of the daphnids (Figure 6). After homogenization, a 600 µL aliquot of 10:9 methanol: water (*v*/*v*) was added to the mixture (Figure 6). The samples were then sonicated for 5 min to precipitate proteins (Figure 6). Then, a 400 µL aliquot of chloroform was added to the samples, and these were manually agitated for 1 min and subsequently allowed to rest for 1 min. The tubes were then centrifuged for 5 min at 4 °C and at 12,000 rpm using an Eppendorf 5804 R centrifuge (Mississauga, ON, Canada). The extraction procedure was repeated three times, and then the 200 µL of the aqueous phase was filtered using a 0.2 µm pore size, polypropylene Mini-UniPrep syringeless filter (GE Healthcare UK, Buckinghamshire, UK, Figure 6). Aliquots of 190 µL of the aqueous phase containing polar metabolites were placed into 2 mL amber chromatography vials, and then 10 µL of a 2000 mg/L stock mixture of isotopically labeled internal standards (glycine-d_2_ (98% purity, Cambridge Isotope Laboratories, Andover, MA, USA), methionine-d_3_ (98% purity, Cambridge Isotope Laboratories, Andover, MA, USA), phenyl-d_5_-alanine (98%, Sigma Aldrich, Mississauga, ON, Canada) and acyclovir (98% purity, Sigma Aldrich, Mississauga, ON, Canada)) in 1:1 methanol: water was added prior to analysis by LC-MS/MS (Figure 6).

Data acquisition was performed using an Agilent 1260 LC system coupled to a 6420A triple quadrupole MS. The LC was equipped with an Ultra Aqueous C_18_ Column (3 µm × 100 mm × 4.6 mm). A gradient elution using both water and acetonitrile with 0.1% formic acid was used as mobile phase during the analysis. Ions were detected in the mass spectrometer using both positive and negative polarities and multiple reaction monitoring, details of which can be found in the Supplementary Materials (Table S8). The sample extracts were calibrated using external standards for 51 metabolites (Supplementary Materials Table S8). Four internal standards (glycine-d_2_, methionine-d_3_, phenyl-d_5_-alanine and acyclovir) were used to confirm the ionization efficiency of the external standards and the metabolites in the samples (Supplementary Materials Table S8). Calibration curves and the calculation of sample concentration were performed using the Mass Hunter Quantitative Analysis program (Agilent Technologies, Mississauga, ON, Canada).

### 4.5. Data Processing and Pathway Analysis

The concentration of metabolites in the *Daphnia* extracts was calculated using generated calibration curves from external standards and then subject to further analysis of the data [53,88]. Absolute metabolite concentrations were imported into MetaboAnalystR (Xia Lab, Montreal, QC, Canada), where the data were filtered to remove outliers based on the interquartile range [58,89,90]. Values below the limit of quantification and those removed as outliers were replaced using half the minimum positive integer to represent the limit of quantification in the data frame for a given metabolite [58,91,92]. Individual metabolite data were normalized using the sum of the total concentrations for each sample using MetaboAnalystR [89,93]. The normalized concentration data points were then scaled using the autoscaling function included in the MetaboAnalystR package [89,93]. The methods previously described have been used across metabolomics experiments with varying contaminants and target organisms [51,53,57,94].

Following normalization of the data, statistical analyses were performed using MetaboAnalystR [93]. The data were first analyzed using multivariate statistics: an unsupervised principal component analysis (PCA). PCA was performed using MetaboAnalyst (version 4.0), which generated a matrix of scores for the top 14 principal components. The scores values were exported into Microsoft Excel, and averaged scores plots (*n* = 12) were generated using OriginPro 8 (version 8E, OriginLab, Northampton, MA, USA). A two-tailed equal variance *t*-test was employed to establish a statistically significant separation between experimental groups. In addition to PCA, partial least squares discriminant analysis (PLS-DA) was also used to assess the separation between experimental groups, and averaged PLS-DA plots were generated as described for PCA. Additional statistical analyses performed include a two-way analysis of variance (ANOVA) where significance was established using *p* < 0.05 [95]. Metabolite percent changes were calculated using the average metabolite concentration of the compound exposure group relative to the average of the unexposed control group [96]. The statistical significance of the changes was determined using a *t*-test (two tailed, equal variance, *p* ≤ 0.05). Along with MetaboAnalystR, R package “ggplot2” was used to generate figures using the RStudio interface (R version 3.6.3, RStudio, Boston, MA, USA) [89,97,98].

Using the pre-processing and normalization methods previously described, the metabolite concentrations were subject to pathway analysis using MetaboAnalyst [58]. The global test algorithm was applied to the KEGG pathway libraries to uncover pathways in *D. magna*. The pathway library for *Drosophila melanogaster* was used as a reference organism [99]. *D. melanogaster* is a common laboratory test insect, and of the reference organisms listed in the pathway libraries, *D. magna* shares more characteristics with *D. melanogaster* than other organisms with known pathways. Pathway analysis allowed for a rapid determination of which biochemical pathways were significantly impacted during sub-lethal exposure to select halogenated acetic acids (DCAA, TCAA, and DBAA).

## 5. Conclusions

Here, we assessed how the acute sub-lethal exposure of three different types of HAAs (DCAA, TCAA and DBAA) altered the polar metabolite profile of *D. magna*. This study has revealed for the first time that DCAA, TCAA and DBAA, despite having different environmental chemistry, invoked a similar response in *D. magna*. Several changes in metabolites were observed, but nucleosides, adenosine, inosine and guanosine were consistently and significantly downregulated with DCAA, TCAA and DBAA exposures. These metabolites are important intermediates in the purine metabolism pathway, and their downregulation perturbed this metabolic pathway. These results, coupled with associated metabolic pathways, collectively point toward energy impairment. As such, the mode of action of DCAA, TCAA and DBAA stems from energy impairment due to disruptions occurring within the TCA cycle and purine metabolism pathways. Interestingly, the exposure to a range of concentrations of HAAs led to a non-monotonic response, suggesting the lack of a linear exposure to response relationship at the molecular level. Based on these observations, it is likely that the contaminants exhibit an HAA class-based mode of action rather than a unique metabolic perturbation for each type of HAA (chlorinated versus brominated). Further studies are warranted to test other types of HAAs to assess the extent of the metabolic disruption observed and confirm the proposed bioindicators, which are the metabolites that responded consistently to exposure, as observed in this study. Additional studies should also evaluate changes in metabolites using a time-course response approach which may provide further insight into how HAAs invoke perturbations to *D. magna* at sub-lethal and environmentally relevant concentrations.

## Figures and Tables

**Table 1:** Summary of pathway analysis perturbations with halogenated acetic acids (HAA) exposure.

Compound	Exposure Concentration (mg/L)	Impacted Pathway	*p* Value
DCAA	20.65	The citric acid (TCA) cycle	0.0761
		Purine metabolism	0.0828
TCAA	8.86	Nicotinate and nicotinamide metabolism Ubiquinone and other terpenoid-quinone biosynthesis Phenylalanine, tyrosine and tryptophan biosynthesis Phenylalanine metabolism Valine, leucine and isoleucine degradation Valine, leucine and isoleucine biosynthesis Pantothenate and CoA biosynthesis Tyrosine metabolism The citric acid (TCA) cycle Histidine metabolism Glycerophospholipid metabolism Thiamine metabolism Aminoacyl-tRNA biosynthesis Butanoate metabolism Cysteine and methionine metabolism	≤0.10
DBAA	7.45	Thiamine metabolism	0.0536
		Tyrosine metabolism	0.0571
		The citric acid (TCA) cycle	0.0840
	14.91	Purine metabolism	0.0810

**Table 2:** Calculated 48-h LC_50_ values for dichloroacetic acid (DCAA), trichloroacetic acid (TCAA) and dibromoacetic acid (DBAA). Sub-lethal toxicities above were obtained using the Environment Canada Biological Test method for *Daphnia magna*. Literature LC_50_ measurements were obtained from Fisher et al. [29].

Contaminant	Calculated 48 h LC_50_ (mg/L)	Literature LC_50_ (mg/L)
Dichloroacetic acid (DCAA)	206.5 ± 25.1	−
Trichloroacetic acid (TCAA)	354.3 ± 22.7	249.5 ^1^
Dibromoacetic acid (DBAA)	298.1 ± 24.7	254.4 ^2^

^1^ TCAA measurements were based on a 21-day chronic LC_50_; ^2^ DBAA measurements were based on a 96-hour acute LC_50_.

## Data Availability

The data presented in this study are available in the article and in the Supplementary Materials.

## Supplementary Materials

The following are available online at https://www.mdpi.com/2218-1989/11/2/100/s1: Table S1: GC-MS parameters used for HAAs detection, Table S2: GC-MS HAA retention times, Table S3: Parameters for the detection of HAAs using tandem mass spectrometry (MS/MS), Table S4: Measured exposure concentrations and standard deviation for DCAA following analysis using LC-MS/MS, Table S5: Measured exposure concentrations and standard deviation for TCAA following analysis using LC-MS/MS, Table S6: Measured exposure concentrations for DBAA following analysis using LC-MS/MS, Table S7: Measured exposure concentrations for control groups following analysis using LC-MS/MS, Table S8: Metabolite class, retention time, MRM and associated internal standards, Figure S1: Averaged (*n* = 12) principal component analysis (PCA) and partial least squares—discriminant analysis (PLS-DA) score plots comparing variation in the metabolic profile across exposure groups to the control for components 1 and 2, and 3 and 4. Figure S2: (A) PLS-DA loadings figure demonstrates the VIP and the relative concentration of the metabolites following DCAA exposure in each corresponding group of the study. (B) ANOVA of metabolite concentrations, inosine (1) and guanosine (2), are statistically significant from the control group after exposure to DCAA (*p* < 0.05). Figure S3: Analysis of averaged metabolite concentrations (*n* = 12) where 0 is the average metabolite concentration across all treatment groups (DCAA exposure and control). Figure S4: Averaged (*n* = 12) metabolite percent changes relative to control. Figure S5: Pearson correlation plot for DCAA exposure which demonstrates the correlation between metabolites measured in *D. magna* extract. Figure S6: (A) PLS-DA loadings figure demonstrates the VIP and the relative concentration of the metabolites following TCAA exposure in each corresponding group of the study. (B) ANOVA of metabolite concentrations, inosine (1) is statistically significant compared to the control group after exposure to TCAA (*p <* 0.05). Figure S7: Analysis of averaged metabolite concentrations (*n* = 12) where 0 is the average metabolite concentration across all treatment groups (TCAA exposure and control). Figure S8: Averaged (*n* = 12) metabolite percent changes following sub-lethal exposure to TCAA relative to control. Figure S9: Pearson correlation plot for TCAA exposure which demonstrates the correlation between metabolites measured in *D. magna* extract. Figure S10: (A) PLS-DA loadings figure demonstrates the VIP and the relative concentration of the metabolites following DBAA exposure in each corresponding group of the study. (B) ANOVA of metabolite concentrations, adenosine (1), inosine (2) and guanosine (3) is statistically significant compared to the control group after exposure to DBAA (*p <* 0.05). Figure S11: Analysis of averaged metabolite concentrations (*n* = 12) where 0 is the average metabolite concentration across all treatment groups (DBAA exposure and control). Figure S12: Averaged (*n* = 12) metabolite percent change following sub-lethal exposure to DBAA relative to control. Figure S13: Pearson correlation plot for TCAA exposure which demonstrates the correlation between metabolites measured in *D. magna* extract. Figure S14: MetaboAnalyst pathway analysis for sub-lethal exposure of *D. magna* to DCAA at 20.65 mg/L. Statistical significance (*p* < 0.10) denoted asterisk (*). Figure S15: MetaboAnalyst pathway analysis for sub-lethal exposure of *D. magna* to TCAA at 8.86 mg/L. Statistical significance (*p* < 0.10) denoted by asterisk (*). Figure S16: MetaboAnalyst pathway analysis for sub-lethal exposure of *D. magna* to DBAA at 7.45 mg/L. Statistical significance (*p* < 0.10) denoted by asterisk (*). Figure S17: MetaboAnalyst pathway analysis for sub-lethal exposure of *D. magna* to DBAA at 7.45 mg/L. Statistical significance (*p* < 0.10) denoted by asterisk (*).

## References

[1] Krasner S.W., Weinberg H.S., Richardson S.D., Pastor S.J., Chinn R., Sclimenti M.J., Onstad G.D., Thruston A.D. (2006). Occurrence of a New Generation of Disinfection Byproducts†. Environ. Sci. Technol..

[2] Diehl A.C., Speitel G.E., Symons J.M., Krasner S.W., Hwang C.J., Barrett S.E. (2000). DBP formation during chloramination. J. Am. Water Work. Assoc..

[3] Selçuk H., Meric C.B.O.A.S., Nikolaou A.D., Bekbolet M. (2011). A comparative study on the control of disinfection by-products (DBPs) and toxicity in drinking water. Desalination Water Treat..

[4] Park K.-Y., Choi S.-Y., Lee S.-H., Kweon J.-H., Song J.-H. (2016). Comparison of formation of disinfection by-products by chlorination and ozonation of wastewater effluents and their toxicity to Daphnia magna. Environ. Pollut..

[5] Richardson S.D., Plewa M.J., Wagner E.D., Schoeny R., DeMarini D.M. (2007). Occurrence, genotoxicity, and carcinogenicity of regulated and emerging disinfection by-products in drinking water: A review and roadmap for research. Mutat. Res. Mutat. Res..

[6] Liu W., Cheung L.-M., Yang X., Shang C. (2006). THM, HAA and CNCl formation from UV irradiation and chlor(am)ination of selected organic waters. Water Res..

[7] Liu W., Zhao Y., Chow C.W., Wang D. (2011). Formation of disinfection byproducts in typical Chinese drinking water. J. Environ. Sci..

[8] Mattei D., Cataudella S., Mancini L., Tancioni L., Migliore L. (2006). Tiber River Quality in the Stretch of a Sewage Treatment Plant: Effects of River Water or Disinfectants to Daphnia and Structure of Benthic Macroinvertebrates Community. Water Air Soil Pollut..

[9] Delinsky A.D., Delinsky D.C., Muralidhara S., Fisher J.W., Bruckner J.V., Bartlett M. (2005). Analysis of dichloroacetic acid in rat blood and tissues by hydrophilic interaction liquid chromatography with tandem mass spectrometry. Rapid Commun. Mass Spectrom..

[10] Uden P.C., Miller J.W. (1983). Chlorinated acids and chloral in drinking water. J. Am. Water Work. Assoc..

[11] DeAngelo A.B. (1996). The carcinogenicity of dichloroacetic acid in the male fischer 344 rat. Toxicology.

[12] Karnes J.B., Usatine R.P. (2014). Management of External Genital Warts. Am. Fam. Physician.

[13] Beutner K.R., Spruance S.L., Hougham A.J., Fox T.L., Owens M.L., Douglas J.M. (1998). Treatment of genital warts with an immune-response modifier (imiquimod). J. Am. Acad. Dermatol..

[14] Bowden D.J., Clegg S.L., Brimblecombe P. (1998). The Henry’s Law Constant of Trichloroacetic Acid. Water Air Soil Pollut..

[15] Bowden D.J., Clegg S.L., Brimblecombe P. (1998). The Henry’s Law Constants of the Haloacetic Acids. J. Atmos. Chem..

[16] Hanson M.L., Sibley P.K., Mabury S.A., Solomon K.R., Muir D.C. (2002). Trichloroacetic acid (TCA) and trifluoroacetic acid (TFA) mixture toxicity to the macrophytes Myriophyllum spicatum and Myriophyllum sibiricum in aquatic microcosms. Sci. Total Environ..

[17] Ellis D.A., Hanson M.L., Sibley P.K., Shahid T., Fineberg N.A., Solomon K.R., Muir D.C.G., Mabury S.A. (2001). The fate and persistence of trifluoroacetic and chloroacetic acids in pond waters. Chemosphere.

[18] Fetzner S. (1998). Bacterial dehalogenation. Appl. Microbiol. Biotechnol..

[19] Bayless W., Andrews R.C. (2007). Biodegradation of six haloacetic acids in drinking water. J. Water Health.

[20] Scott B.F., MacTavish D., Spencer C., Strachan W.M.J., Muir D.C.G. (2000). Haloacetic Acids in Canadian Lake Waters and Precipitation. Environ. Sci. Technol..

[21] Melo A., Ferreira C., Ferreira I.M., Mansilha C.R. (2019). Acute and chronic toxicity assessment of haloacetic acids using Daphnia magna. J. Toxicol. Environ. Health Part A.

[22] Hanson M.L., Solomon K.R. (2004). Haloacetic acids in the aquatic environment. Part I: Macrophyte toxicity. Environ. Pollut..

[23] Berends A., Boutonnet J.C., De Rooij C.G., Thompson R.S. (1999). Toxicity of trifluoroacetate to aquatic organisms. Environ. Toxicol. Chem..

[24] Fitzsimmons P.N., Hoffman A.D., Lien G.J., Hammermeister D.E., Nichols J.W. (2009). Kinetics and effects of dichloroacetic acid in rainbow trout. Aquat. Toxicol..

[25] Hanson M.L., Sibley P.K., A Mabury S., Muir D.C., Solomon K.R. (2003). Field level evaluation and risk assessment of the toxicity of dichloroacetic acid to the aquatic macrophytes Lemna gibba, Myriophyllum spicatum, and Myriophyllum sibiricum. Ecotoxicol. Environ. Saf..

[26] Toussaint M.W., Brennan L.M., Rosencrance A.B., Dennis W.E., Hoffmann F.J., Gardner H.S. (2001). Acute Toxicity of Four Drinking Water Disinfection By-Products to Japanese Medaka Fish. Bull. Environ. Contam. Toxicol..

[27] Williams F.E., Sickelbaugh T.J., Hassoun E. (2006). Modulation by ellagic acid of DCA-induced developmental toxicity in the zebrafish (Danio rerio). J. Biochem. Mol. Toxicol..

[28] Hassoun E., Kariya C., Williams F.E. (2005). Dichloroacetate-induced developmental toxicity and production of reactive oxygen species in zebrafish embryos. J. Biochem. Mol. Toxicol..

[29] Fisher D., Yonkos L., Ziegler G., Friedel E., Burton D. (2014). Acute and chronic toxicity of selected disinfection byproducts to Daphnia magna, Cyprinodon variegatus, and Isochrysis galbana. Water Res..

[30] Giller S., Le Curieux F., Erb F., Marzin D. (1997). Comparative genotoxicity of halogenated acetic acids found in drinking water. Mutagenesis.

[31] Rizzo L., Belgiorno V., Gallo M., Meric S. (2005). Removal of THM precursors from a high-alkaline surface water by enhanced coagulation and behaviour of THMFP toxicity on D. magna. Desalination.

[32] Andrews J., Nichols H., Schmid J., Mole L., Hunter E., Klinefelter G. (2004). Developmental toxicity of mixtures: The water disinfection by-products dichloro-, dibromo- and bromochloro acetic acid in rat embryo culture. Reprod. Toxicol..

[33] Smith M.K., Randall J.L., Read E.J., Stober J.A. (1992). Developmental toxicity of dichloroacetate in the rat. Teratology.

[34] Smith M.K., Randall J.L., Read E.J., Stober J.A. (1989). Teratogenic activity of trichloroacetic acid in the rat. Teratology.

[35] Epstein D.L., Nolen G.A., Randall J.L., Christ S.A., Read E.J., Stober J.A., Smith M.K. (1992). Cardiopathic effects of dichloroacetate in the fetal long-evans rat. Teratology.

[36] Bull R.J., Orner G.A., Cheng R.S., Stillwell L., Stauber A.J., Sasser L.B., Lingohr M.K., Thrall B.D. (2002). Contribution of Dichloroacetate and Trichloroacetate to Liver Tumor Induction in Mice by Trichloroethylene. Toxicol. Appl. Pharmacol..

[37] Echigo S., Itoh S., Natsui T., Araki T., Ando R. (2004). Contribution of brominated organic disinfection by-products to the mutagenicity of drinking water. Water Sci. Technol..

[38] Yang M., Zhang X. (2013). Comparative Developmental Toxicity of New Aromatic Halogenated DBPs in a Chlorinated Saline Sewage Effluent to the Marine PolychaetePlatynereis dumerilii. Environ. Sci. Technol..

[39] Lambert V., Hansen S., Schoumacher M., LeComte J., Leenders J., Hubert P., Herfs M., Blacher S., Carnet O., Yip C. (2020). Pyruvate dehydrogenase kinase/lactate axis: A therapeutic target for neovascular age-related macular degeneration identified by metabolomics. J. Mol. Med..

[40] Pettit F.H., Pelley J.W., Reed L.J. (1975). Regulation of pyruvate dehydrogenase kinase and phosphatase by acetyl-CoA/CoA and NADH/NAD ratios. Biochem. Biophys. Res. Commun..

[41] Law J.M., Lopez L., DeAngelo A.B. (1998). Hepatotoxicity of the drinking water disinfection by-product, dichloroacetic acid, in the medaka small fish model. Toxicol. Lett..

[42] Lee B.-Y., Choi B.-S., Kim M.-S., Park J.C., Jeong C.-B., Han J., Lee J.-S. (2019). The genome of the freshwater water flea Daphnia magna: A potential use for freshwater molecular ecotoxicology. Aquat. Toxicol..

[43] Smirnov N.N. (2014). Physiology of the Cladocera.

[44] Sarma S.S.S., Nandini S. (2006). Review of Recent Ecotoxicological Studies on Cladocerans. J. Environ. Sci. Health Part B.

[45] Han G.H., Hur H.G., Kim S.D. (2006). Ecotoxicological Risk of Pharmaceuticals from Wastewater Treatment Plants in Korea: Occurrence and Toxicity to Daphnia Magna. Environ. Toxicol. Chem..

[46] Jansen M., Coors A., Stoks R., De Meester L. (2011). Evolutionary ecotoxicology of pesticide resistance: A case study in Daphnia. Ecotoxicology.

[47] Woermann M., Sures B. (2020). Ecotoxicological effects of micropollutant-loaded powdered activated carbon emitted from wastewater treatment plants on Daphnia magna. Sci. Total Environ..

[48] Taylor N.S., Weber R.J.M., Southam A.D., Payne T.G., Hrydziuszko O., Arvanitis T.N., Viant M.R. (2009). A new approach to toxicity testing in Daphnia magna: Application of high throughput FT-ICR mass spectrometry metabolomics. Metabolomics.

[49] Taylor N.S., Weber R.J.M., White T.A., Viant M.R. (2010). Discriminating between Different Acute Chemical Toxicities via Changes in the Daphnid Metabolome. Toxicol. Sci..

[50] Nagato E.G., Simpson A.J., Simpson M.J. (2016). Metabolomics reveals energetic impairments in Daphnia magna exposed to diazinon, malathion and bisphenol-A. Aquat. Toxicol..

[51] Jeong T.-Y., Simpson M.J. (2019). Daphnia magna metabolic profiling as a promising water quality parameter for the biological early warning system. Water Res..

[52] Jeong T.-Y., Simpson M.J. (2020). Time-dependent biomolecular responses and bioaccumulation of perfluorooctane sulfonate (PFOS) in Daphnia magna. Comp. Biochem. Physiol. Part D Genom. Proteom..

[53] Jeong T.-Y., Simpson M.J. (2019). Reproduction Stage Differentiates the Time-Course Regulation of Metabolites in Daphnia magna. Environ. Sci. Technol..

[54] Jeong T.-Y., Simpson M.J. (2020). Reproduction stage specific dysregulation of Daphnia magna metabolites as an early indicator of reproductive endocrine disruption. Water Res..

[55] Saccenti E., Hoefsloot H.C.J., Smilde A.K., Westerhuis J.A., Hendriks M.M.W.B. (2014). Reflections on univariate and multi-variate analysis of metabolomics data. Metabolomics.

[56] Worley B., Powers R. (2012). Multivariate Analysis in Metabolomics. Curr. Metab..

[57] Kapoore R.V., Vaidyanathan S. (2016). Towards quantitative mass spectrometry-based metabolomics in microbial and mammalian systems. Philos. Trans. R. Soc. A Math. Phys. Eng. Sci..

[58] Chong J., Soufan O., Li C., Caraus I., Li S., Bourque G., Wishart D.S., Xia J. (2018). MetaboAnalyst 4.0: Towards more trans-parent and integrative metabolomics analysis. Nucleic Acids Res..

[59] Machado A.A.D.S., Zarfl C., Rehse S., Kloas W. (2017). Low-Dose Effects: Nonmonotonic Responses for the Toxicity of a Bacillus thuringiensis Biocide to Daphnia magna. Environ. Sci. Technol..

[60] Kim J., Lee S., Kim C.-M., Seo J., Park Y., Kwon D., Lee S.-H., Yoon T.-H., Choi K. (2014). Non-monotonic concentration–response relationship of TiO2 nanoparticles in freshwater cladocerans under environmentally relevant UV-A light. Ecotoxicol. Environ. Saf..

[61] Wagner N.D., Helm P.A., Simpson A.J., Simpson M.J. (2019). Metabolomic responses to pre-chlorinated and final effluent wastewater with the addition of a sub-lethal persistent contaminant in Daphnia magna. Environ. Sci. Pollut. Res..

[62] Kariuki M.N., Nagato E.G., Lankadurai B.P., Simpson A.J., Simpson M.J. (2017). Analysis of Sub-Lethal Toxicity of Perfluorooctane Sulfonate (PFOS) to Daphnia magna Using 1H Nuclear Magnetic Resonance-Based Metabolomics. Metabolities.

[63] Kakuda T.N. (2000). Pharmacology of nucleoside and nucleotide reverse transcriptase inhibitor-induced mitochondrial toxicity. Clin. Ther..

[64] Oikawa T.G., Smith M. (1966). Nucleotides in the Encysted Embryos of Daphnia magna. Biochemistry.

[65] Gilmour S.J., Warner A.H. (1978). The Presence of Guanosine 5’-Diphospho-5’qguanosine and Guanosine 5’-Triphospho-5’-adenosine in Brine Shrimp Embryos. J. Biol. Chem..

[66] Pedley A.M., Benkovic S.J. (2017). A New View into the Regulation of Purine Metabolism: The Purinosome. Trends Biochem. Sci..

[67] Orgel L.E. (1986). RNA catalysis and the origins of life. J. Theor. Biol..

[68] Müller-Hill B. (2006). What is life? The paradigm of DNA and protein cooperation at high local concentrations. Mol. Microbiol..

[69] Michalak E.M., Burr M.L., Bannister A.J., Dawson M.A. (2019). The roles of DNA, RNA and histone methylation in ageing and cancer. Nat. Rev. Mol. Cell Biol..

[70] Kanehisa M., Sato Y., Kawashima M., Furumichi M., Tanabe M. (2016). KEGG as a reference resource for gene and protein annotation. Nucleic Acids Res..

[71] Campos B., Garcia-Reyero N., Rivetti C., Escalon L., Habib T., Tauler R., Tsakovski S., Piña B., Barata C. (2013). Identification of Metabolic Pathways in Daphnia magna Explaining Hormetic Effects of Selective Serotonin Reuptake Inhibitors and 4-Nonylphenol Using Transcriptomic and Phenotypic Responses. Environ. Sci. Technol..

[72] Yan L.-J., Levine R.L., Sohal R.S. (1997). Oxidative damage during aging targets mitochondrial aconitase. Proc. Natl. Acad. Sci. USA.

[73] Beinert H., Kennedy A.M.C., Stout§ C.D. (1996). Aconitase as Iron−Sulfur Protein, Enzyme, and Iron-Regulatory Protein. Chem. Rev..

[74] Zurita J.L., Jos A., Cameán A.M., Salguero M., López-Artíguez M., Repetto G. (2007). Ecotoxicological evaluation of sodium fluoroacetate on aquatic organisms and investigation of the effects on two fish cell lines. Chemosphere.

[75] Goyer A. (2010). Thiamine in plants: Aspects of its metabolism and functions. Phytochemistry.

[76] Allen S., Zilles J.L., Downs D.M. (2002). Metabolic Flux in Both the Purine Mononucleotide and Histidine Biosynthetic Pathways Can Influence Synthesis of the Hydroxymethyl Pyrimidine Moiety of Thiamine in Salmonella enterica. J. Bacteriol..

[77] Rapala-Kozik M., Kowalska E., Ostrowska K. (2008). Modulation of thiamine metabolism in Zea mays seedlings under conditions of abiotic stress. J. Exp. Bot..

[78] Mayr J.A., Freisinger P., Schlachter K., Rolinski B., Zimmermann F.A., Scheffner T., Haack T.B., Koch J., Ahting U., Prokisch H. (2011). Thiamine Pyrophosphokinase Deficiency in Encephalopathic Children with Defects in the Pyruvate Oxidation Pathway. Am. J. Hum. Genet..

[79] Curtius H.-C., Völlmin J., Baerlocher K. (1972). The use of deuterated phenylalanine for the elucidation of the phenylalanine-tyrosine metabolism. Clin. Chim. Acta.

[80] Gertsman I., Gangoiti J.A., Nyhan W.L., A Barshop B. (2015). Perturbations of tyrosine metabolism promote the indolepyruvate pathway via tryptophan in host and microbiome. Mol. Genet. Metab..

[81] Ontario Ministry of the Environment (2012). Daphnia Magna Culturing.

[82] Ontario Ministry of the Environment (2012). Algae Culturing for Use as Food.

[83] Environment Canada (2000). Biological Test Method: Reference Method for Determining Acute Lethality of Effluents to Daphnia Magna.

[84] Robertson J.L., Russell R.M., Savin N.E. (1980). POLO: A User’s Guide to Probit or LOgit Analysis.

[85] Xie Y. (2001). Analyzing Haloacetic Acids Using Gas Chromatography/Mass Spectrometry. Water Res..

[86] Meng L., Wu S., Ma F., Jia A., Hu J. (2010). Trace determination of nine haloacetic acids in drinking water by liquid chromatography–electrospray tandem mass spectrometry. J. Chromatogr. A.

[87] Luo Q., Wang D., Wei Z., Wang Z. (2013). Optimized chromatographic conditions for separation of halogenated acetic acids by ultra-performance liquid chromatography–electrospray ionization-mass spectrometry. J. Chromatogr. A.

[88] Wu Y., Li L. (2016). Sample normalization methods in quantitative metabolomics. J. Chromatogr. A.

[89] Pang Z., Chong J., Li S., Xia J. (2020). MetaboAnalystR 3.0: Toward an Optimized Workflow for Global Metabolomics. Metabolities.

[90] Castillo S., Gopalacharyulu P., Yetukuri L., Orešič M. (2011). Algorithms and tools for the preprocessing of LC–MS metabolomics data. Chemom. Intell. Lab. Syst..

[91] Chong J., Wishart D.S., Xia J. (2019). Using MetaboAnalyst 4.0 for Comprehensive and Integrative Metabolomics Data Analysis. Curr. Protoc. Bioinform..

[92] Sun X., Weckwerth W. (2012). COVAIN: A toolbox for uni- and multivariate statistics, time-series and correlation network analysis and inverse estimation of the differential Jacobian from metabolomics covariance data. Metabolomics.

[93] Chong J., Xia J. (2018). MetaboAnalystR: An R package for flexible and reproducible analysis of metabolomics data. Bioinformatics.

[94] Rusilowicz M., Dickinson M., Charlton A., O’Keefe S., Wilson J. (2016). A batch correction method for liquid chromatography–mass spectrometry data that does not depend on quality control samples. Metabolomics.

[95] Flores-Valverde A.M., Horwood J., Hill E.M. (2010). Disruption of the Steroid Metabolome in Fish Caused by Exposure to the Environmental Estrogen 17α-Ethinylestradiol. Environ. Sci. Technol..

[96] Southam A.D., Lange A., Hines A., Hill E.M., Katsu Y., Iguchi T., Tyler C.R., Viant M.R. (2011). Metabolomics Reveals Target and Off-Target Toxicities of a Model Organophosphate Pesticide to Roach (Rutilus Rutilus): Implications for Biomonitoring. Environ. Sci. Technol..

[97] Wickham H. (2016). ggplot2: Elegant Graphics for Data Analysis.

[98] R Core Team (2020). R: A Language Environment for Statistical Computing.

[99] Kovacevic V., Simpson A.J., Simpson M.J. (2016). 1H NMR-based metabolomics of Daphnia magna responses after sub-lethal exposure to triclosan, carbamazepine and ibuprofen. Comp. Biochem. Physiol. Part D Genom. Proteom..

