# Supplementary material for: Targeted Metabolomic Assessment of the Sub-Lethal Toxicity of Halogenated Acetic Acids (HAAs) to Daphnia magna
Source: Metabolites. 2021 Feb 10;11(2):100. doi: 10.3390/metabo11020100 (PMC7916598; doi:10.3390/metabo11020100)
Supplement: Supplementary file 1 [file metabolites-11-00100-s001.pdf]

Supplementary Materials

Targeted metabolomic assessment of sub-lethal toxicity of halogenated acetic acids (HAAs) to *Daphnia magna*

Lisa M. Labine [1,2] and Myrna J. Simpson [1,2,*]

1 Department of Chemistry University of Toronto, 80 St. George St., Toronto, ON, M5S 3H6
2 Environmental NMR Centre and Department of Physical and Environmental Sciences, University of Toronto Scarborough, 1265 Military Trail, Toronto, ON, M1C 1A4.
* Correspondence: myrna.simpson@utoronto.ca ; Tel.: +1-416-287-7234

1. Lethal concentration (LC_{50}) determination for halogenated acetic acids (HAAs)

1.1. HAAs preparation for LC_{50} experiments

Concentrations of dichloroacetic acid (DCAA), trichloroacetic acid (TCAA), and dibromoacetic acid (DBAA) were confirmed before and after the lethal concentration (LC_{50}) test by sampling 30 mL aliquots from each concentration replicate. The aliquots were filtered through a 0.2 μm PTFE membrane filter (Target2, Thermo Scientific, Rockwood, TN) to remove algae from the samples. The filtered media was placed into 50 mL glass tubes with 12 g of sodium sulfate (Fisher Scientific, Fair Lawn, NJ; 99.8% purity) and then acidified with 2 mL of 18 M sulfuric acid (Fisher Scientific, Fair Lawn, NJ; < 95% purity). To the acidified media, 3 mL of methyl tert-butyl ether (MTBE; Alfa Aesar, Ward Hill, MA; 99% purity) was added. The tubes were then manually shaken for 1 min. For derivatization prior to gas chromatography (GC)-mass spectrometry (MS), the organic phase was removed and transferred into 10 mL vials where 1 mL of 10% (*v/v*) sulfuric acid solution in methanol (Fisher Scientific, Fair Lawn, NJ; 99.9% purity) was added. The samples were then heated to 50 °C for 2 h. The samples were then transferred to 20 mL vials and 4 mL of a 0.704 M sodium sulfate solution was added to remove any underivatized compounds from the organic phase. The organic phase was transferred into 2mL chromatography vials for analysis of the LC_{50} concentration of the halogenated acetic acids (HAAs).

1.1. Quantification of HAAs by gas chromatography – mass spectrometry (GC-MS)

Analysis of the LC_{50} HAA concentrations was performed using gas chromatography (GC, Agilent Technologies, 7890B) hyphenated with a mass spectrometer (MS, Agilent Technologies, 5977B) using a previously published method [85]. The oven temperature was first held at 35 °C for 10 min followed by a ramping to 75 °C at a rate of 5 °C/min. The temperature was then held constant for 15 min, and then increased to 185 °C at a rate of 25 °C/min and held for one minute. The total temperature program lasted 38.4 min and all compounds were eluted within the first 20 min of the program. Details of the parameters used in the mass spectrometer are summarized in Table S1 and Table S2. The concentrations of the LC_{50} exposure media were confirmed using the method above for DCAA, TCAA, and DBAA.

Table 1. Gas chromatography - mass spectrometry (GC-MS) parameters used for halogenated acetic acid (HAAs) detection.

Parameter	Details
Carrier Gas	N_2
Injection Temperature	200 °C
Transfer Line Temperature	280 °C
Split	1:10
Ionization	Electron Impact (EI)
Voltage	70 eV
Column	HP-5MS (30m × 0.25mm (I.D.) × 0.25μm film thickness)

Injection Volume	3 µL

Table S2. Gas chromatography-mass spectrometry (GC-MS) HAA retention times.

Compound	Retention Time (min)	Derivatized Compound
Dichloroacetic acid (DCAA)	10.162	Methyl dichloroacetate
Trichloroacetic acid (TCAA)	15.057	Methyl trichloroacetate
Dibromoacetic acid (DBAA)	18.779	Methyl dibromoacetate

2. LC_{50} reference toxicant (NaCl) sample preparation and analysis by ion chromatography (IC)

Concentration of the reference toxicant was also confirmed at the beginning and end of the LC_{50} test, as such aliquots of 30 mL were taken from the exposure media both before and after the 48 h exposure. The aliquots were filtered through a 0.22 µm PTFE membrane filter (Target2, Thermo Scientific, Rockwood, TN) to remove algae and particulate matter from the samples. The samples were then placed into 15 mL tubes and ion chromatography (IC) was used to determine the concentration of chloride anion in the samples to confirm the amount of NaCl (used as a reference during LC_{50} measurements). Anion chromatography was performed using a Metroohm 930 compact IC flux. The chromatography mobile phase was water with 1 mM of $NaHCO_3$ and 3.2 mM of Na_2CO_3 and an ion regeneration solution of water with 100 mM of H_2SO_4. All standards and samples were diluted ten times before the analysis of the anions. The concentrations of the LC_{50} exposure reference toxicant was confirmed using the concentration of chloride in the sample which was calibrated using a standard addition standard curve.

3. *Daphnia* exposure to halogenated acetic acids (HAAs)

3.1. HAAs sub-lethal exposure sample preparation

To confirm the nominal concentration of DCAA, TCAA, and DBAA in the exposure media, aliquots of 3 mL were sampled both before and after the 48 h sub-lethal exposure to *D. magna*. The aliquots were filtered through a 0.22 µm PTFE membrane filter (Target2, Thermo Scientific, Rockwood, TN) to remove algae from the samples. The filtrate was then diluted using ultrapure (<18 MΩ) water so the concentrations of the samples would fall within the linear calibration range for each of the compounds. Once the samples were diluted, they were then transferred into 2 mL amber chromatography vials for analysis.

3.2. LC-MS/MS Analysis for confirming nominal exposure concentrations

3.2.1. Analysis of DCAA in Daphnia exposure media

DCAA concentration measurements were based on the method of Meng et al. [86]. The instrument used for analysis was an Agilent Technologies 1260 Infinity (Agilent Technologies, Mississauga, ON) hyphenated to an Agilent Technologies 6420A triple quadrupole MS (Agilent Technologies, Mississauga, ON). LC separation of DCAA was performed using a Waters Acquity BEH C_8 (2.1 mm × 100 mm × 1.7 µm) column (Waters Corporation; Milford, MA). The column compartment was maintained at 40 °C, and the binary pump operated at a flow rate of 0.225 mL/min. Samples of 10 µL were injected into the column for chromatographic separation which occurred using gradient elution composed of mobile phase A (water with 0.5% acetic acid (*v*/*v*)) and mobile phase B (acetonitrile with 0.1% acetic acid (*v*/*v*)) beginning at a 95:5 solvent composition. The gradient then changed to a 60:40 (A: B) composition over 4 min. The program then increased composition of B to 100% over the next minute. The program was held at 100% B for one minute before returning to the initial composition of 95:5 over 1.5 min. The total program leads to a run time of 7.5 min, with a post equilibration time of 5 min after every sample run. The sample entered the MS where it was negatively ionized using electrospray ionization (ESI) with the source at a temperature of 350 °C and a gas flow rate of 8 L/min and a nebulizer pressure of 40 psi. The voltage of the discharge capillary is 3000 eV. The MS1 and MS2 temperatures were both maintained at 100 °C during the analysis. The

parameters for the fragmentation of the ions can be found in Table S3 along with the information for TCAA and DBAA.

3.2.2. Analysis of TCAA and DBAA in Daphnia exposure media

TCAA and DBAA concentration measurements were based on the method of Luo et al. [87]. The instrument used for analysis was an Agilent Technologies 1260 Infinity (Agilent Technologies, Mississauga, ON) hyphenated to an Agilent Technologies 6420A triple quadrupole MS (Agilent Technologies, Mississauga, ON). The LC separation of DCAA and DBAA was performed using a Waters Acquity BEH C8 (2.1 mm × 100 mm × 1.7 μm) column (Milford, MA). Samples of 10 μL were injected into the column to undergo separation and analysis. The column compartment was maintained at 40 °C, and the binary pump operated at a flow rate of 0.225 mL/min. Chromatographic separation occurred using gradient elution composed of mobile phase A (water with 10mM ammonium formate) and mobile phase B (acetonitrile) beginning at a 40:60 solvent composition. The gradient then changed to a composition of 60:40 (A: B) over 1.5 min. The program then increased the percentage of mobile phase A to a composition of 75:25 (A:B) over 2.5 min. The solvent composition was then increased to 100% mobile phase A over 2 min. Over the next 1.5 min the composition changed to 95:5 (A:B). The total run time of each chromatographic separation was 5 min with a post-equilibration time of 2 min. The sample entered the mass spectrometer where it was negatively ionized using electrospray ionization (ESI) with the source chamber at a temperature of 350 °C, and the voltage of the discharge capillary set to 3000 eV. The gas flow rate was 8 L/min with a nebulizer pressure of 40 psi. During the analysis the temperature of the MS1 and MS2 were maintained at 100 °C. The parameters for the fragmentation of the ions can be found in Table S3 along with the information for DCAA.

Table S3. Parameters for the detection of halogenated acetic acids (HAAs) using tandem mass spectrometry (MS/MS).

**Compound**	**Precursor Ion (M-H$^+$) (*m/z*)**	**Fragmentor**	**Collision Energy**	**Product Ion (*m/z*)**
Dichloroacetic acid (DCAA)	126.9	50 eV	8	83.0
Trichloroacetic acid (TCAA)	160.9	110 eV	5	116.9
Dibromoacetic acid (DBAA)	214.8	50 eV	4	170.0

Table S4. Measured exposure concentrations and standard deviation for dichloroacetic acid (DCAA) following analysis using LC-MS/MS.

**Nominal Concentration (mg/L)**	***Dichloroacetic Acid (DCAA)***	
	Measured DCAA Concentration (mg/L)	
	**0 h**	**48 h**
5.16	5.78 ± 0.17	5.05 ± 0.06
6.88	7.70 ± 0.06	6.89 ± 0.06
10.32	11.65 ± 0.07	10.04 ± 0.08
20.65	22.74 ± 0.07	21.64 ± 0.17

Table S5. Measured exposure concentrations and standard deviation for trichloroacetic acid (TCAA) following analysis using LC-MS/MS.

**Nominal Concentration (mg/L)**	**Trichloroacetic acid (TCAA)**	
	Measured TCAA Concentration (mg/L)	
	**0 h**	**48 h**
8.86	8.81 ± 1.26	8.94 ± 0.05
11.81	11.62 ± 1.07	12.17 ± 0.68

17.72	17.78 ± 1.10	17.45 ± 0.16
35.43	35.06 ± 0.47	35.14 ± 2.24

Table S6. Measured exposure concentrations for dibromoacetic acid (DBAA) following analysis using LC-MS/MS.

Nominal Concentration (mg/L)	Dibromoacetic acid (DBAA)	
	Measured DBAA Concentration (mg/L)	
	**0 h**	**48 h**
7.45	7.38 ± 0.20	7.02 ± 0.08
9.94	10.16 ± 0.42	8.72 ± 0.15
14.91	15.39 ± 0.35	13.57 ± 0.37
29.81	31.70 ± 0.44	28.54 ± 0.67

Table S7. Measured exposure concentrations for control groups following analysis using LC-MS/MS.

Exposure Group	DCAA Concentration (µg/L)	TCAA Concentration (µg/L)	DBAA Concentration (µg/L)
Control – 0 h	nd [a]	nd[a]	nd[a]
Control – 48 h	nd[a]	5.58 ± 7.50	nd[a]

[a]nd = not detected.

Table S8. Metabolite class, retention time, MRM, and associated internal standards.

Metabolite	Class	Retention Time (min)	MRM[1]	Internal Standard (ITSD)[2]
Putrescine	Amino acid derivative	0.845	**89.0 > 68.5**<br>89.0 > 72.0	Glycine-d_2
Spermidine	Polyamine	0.852	146.2 > 72.0<br>**146.0 > 73.2**	Glycine-d_2
Histamine	Neurotransmitter	0.883	112.0 > 68.0<br>112.0 > 83.2<br>**112.0 > 95.1**	Glycine-d_2
Trimethylenediamine	Polyamine	0.866	75.0 > 41.0<br>**75.0 > 58.2**	Glycine-d_2
Ornithine	Amino acid	0.938	**133.0 > 70.0**<br>133.0 > 116.1	Glycine-d_2
Histidine	Amino acid	0.993	**156.1 > 110.1**	Glycine-d_2
Arginine	Amino acid	1.002	**175.1 > 70.2**	Glycine-d_2
Cystine Dimer	Amino acid derivative	1.011	**241.0 > 74.1**	Glycine-d_2
Glucose-6-phosphate	Saccharide derivative	1.028	**259.0 > 79.0**<br>259.0 > 96.9	Glycine-d_2
Serine	Amino acid	1.044	**106.1 > 60.2**	Glycine-d_2
Glycine	Amino acid	1.045	**76.0 > 30.2**	Glycine-d_2
Aspartic acid	Amino acid	1.061	**134.1 > 74.1**	Glycine-d_2
S-adenosylmethionine	Amino acid derivative	1.067	**399.0 > 250.0**	Glycine-d_2
Glutamine	Amino acid	1.080	**147.1 > 84.1**	Glycine-d_2
Lysine	Amino acid	1.080	**147.1 > 84.1**	Glycine-d_2
Homoserine	Amino acid derivative	1.081	120.0 > 56.0<br>**120.0 > 74.2**	Glycine-d_2
Threonine	Amino acid	1.091	**120.1 > 74.2**	Glycine-d_2
Alanine	Amino acid	1.083	**89.9 > 57.0**	Glycine-d_2
GABA	Neurotransmitter	1.092	104.1 > 86.1<br>**104.1 > 87.1**	Glycine-d_2
Glutamic acid	Amino acid	1.099	**148.1 > 84.1**	Glycine-d_2
Hydroxyproline	Amino acid derivative	1.109	132.0 > 68.2<br>**132.0 > 86.1**	Glycine-d_2
Thiamine	Vitamin	1.116	**265.0 > 122.0**<br>265.0 > 144.0	Glycine-d_2
Citrulline	Amino acid	1.126	**176.0 > 70.0**<br>176.0 > 113.0	Glycine-d_2
CMP	Nucleoside	1.201	324.0 > 97.0<br>**324.0 > 112.0**	Acyclovir

Table S8. Continued. Metabolite class, retention time, MRM, and associated internal standards.

Metabolite	Class	Retention Time (min)	MRM[1]	Internal Standard (ITSD)[2]
Acetyllysine	Amino acid derivative	1.221	**189.2 > 84.2**<br>189.2 > 129.1	Glycine-d_2
Carnitine	Polyamine	1.231	162.0 > 60.2<br>**162.0 > 103.0**	Glycine-d_2
Choline	Neurotransmitter	1.234	104.0 > 58.0<br>**104.0 > 60.0**	Glycine-d_2
Proline	Amino acid	1.272	**116.1 > 70.2**	Glycine-d_2
UMP	Nucleoside	1.276	**325.0 > 97.0**<br>325.0 > 112.9	Acyclovir
Malic acid	Carboxylic acid	1.313	133.0 > 70.9<br>**133.0 > 114.8**	Methionine-d_3
AMP	Nucleoside	1.314	**348.0 > 136.0**<br>348.0 > 337.1	Acyclovir
Dopamine	Neurotransmitter	1.326	154.1 > 91.1<br>**154.1 > 137.0**	Methionine-d_3
Valine	Amino acid	1.319	**118.1 > 72.0**	Methionine-d_3
Methionine	Amino acid	1.336	**150.1 > 104.1**	Methionine-d_3
Acetylcholine	Neurotransmitter	1.327	146.1 > 60.1<br>**146.1 > 87.0**	Methionine-d_3
Nicotinic acid	Vitamin	1.851	124.1 > 78.1<br>**124.1 > 80.3**	Methionine-d_3
Citric acid	Carboxylic acid	1.844	191.0 > 86.7<br>**191.0 > 111.0**	Methionine-d_3
GMP	Nucleoside	1.845	364.0 > 97.2<br>**364.0 > 152.0**	Acyclovir
IMP	Nucleoside	1.845	349.0 > 96.8<br>**349.0 > 137.0**	Acyclovir
Asparagine	Amino acid	2.001	**133.1 > 87.0**	Methionine-d_3
Leucine	Amino acid	2.002	**132.1 > 86.2**	Methionine-d_3
Uridine	Nucleoside	2.008	245.0 > 109.4<br>**245.0 > 113.0**	Acyclovir
Isoleucine	Amino acid	2.124	**132.1 > 86.2**	Methionine-d_3
Tyrosine	Amino acid	2.254	**182.1 > 165.0**	Methionine-d_3
Tyramine	Nucleoside	2.330	138.0 > 93.1<br>**138.0 > 121.0**	Methionine-d_3
Adenosine	Nucleoside	2.682	268.0 > 57.0<br>**268.0 > 136.0**	Acyclovir
Inosine	Nucleoside	2.831	269.0 > 57.1<br>**269.0 > 137.0**	Acyclovir

Table S8. continued. Metabolite class, retention time, MRM, and associated internal standards.

Metabolite	Class	Retention Time (min)	MRM[1]	Internal Standard (ITSD)[2]
Guanosine	Nucleoside	2.886	284.0 > 134.5<br>**284.0 > 152.2**	Acyclovir
Phenylalanine	Amino acid	3.269	**166.1 > 120.1**	Phenyl-d_5-alanine
Tryptophan	Amino acid	4.041	**205.1 > 188.1**	Phenyl-d_5-alanine
Riboflavin	Vitamin	4.626	**377.0 > 243.0**<br>377.0 > 359.0	Phenyl-d_5-alanine
Cytidine	Nucleoside	6.878	**244.0 > 83.1**<br>244.0 > 97.0	Acyclovir
Acyclovir	Nucleoside Analogue	2.975	**226.1 > 151.9**	ITSD
Glycine-d_2	Amino acid	1.045	**78.1 > 32.2**	ITSD
Methionine-d_3	Amino acid	1.326	**153.1 > 56.2**	ITSD
Phenyl-d_5-alanine	Amino acid	3.230	**171.1 > 106.1**	ITSD

[1]Multiple reaction monitoring (MRM) in bold used for quantification, secondary MRM was used for qualitative confirmation of the metabolite. [2]Internal standards used to ensure ionization efficiency of external standards and endogenous metabolites of *D. magna*.

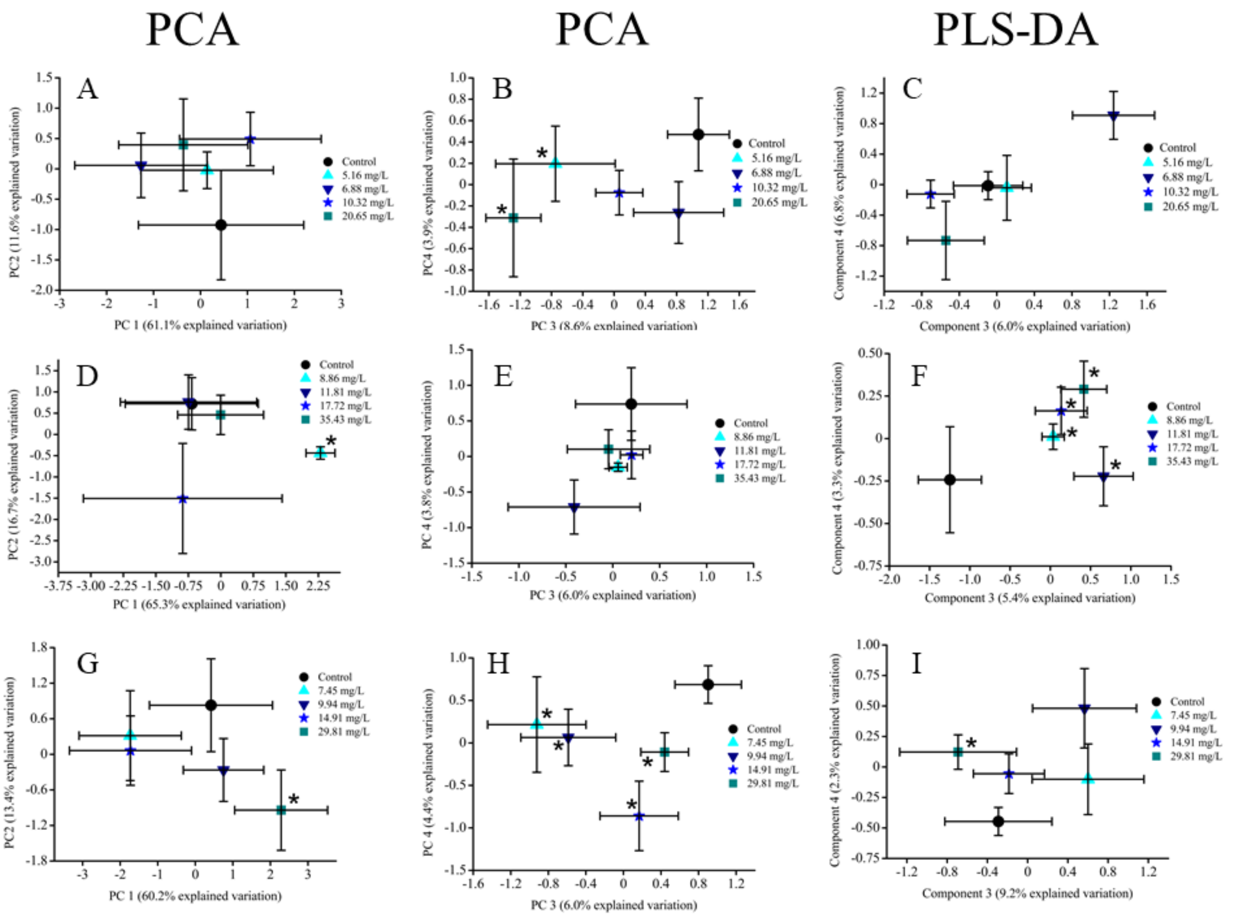

Figure S1. Averaged (n = 12) principal component analysis (PCA) and partial least squares – discriminant analysis (PLS-DA) scores plots comparing variation in the metabolic profile across exposure groups to the control for components 1 and 2, and 3 and 4. Statistical significance ($p < 0.05$) is outlined by the presence of an asterisk (*). A) PCA PC 1 and PC 2 of DCAA exposure, B) PCA PC 3 and PC 4 of DCAA exposure, C) PLS-DA of DCAA exposure, D) PCA PC 1 and PC 2 of TCAA exposure, E) PCA PC 3 and PC 4 of TCAA exposure, F) PLS-DA of TCAA exposure, G) PCA PC 1 and PC 2 of DBAA exposure, H) PCA PC 3 and PC 4 of DBAA exposure, I) PLS-DA of DBAA exposure.

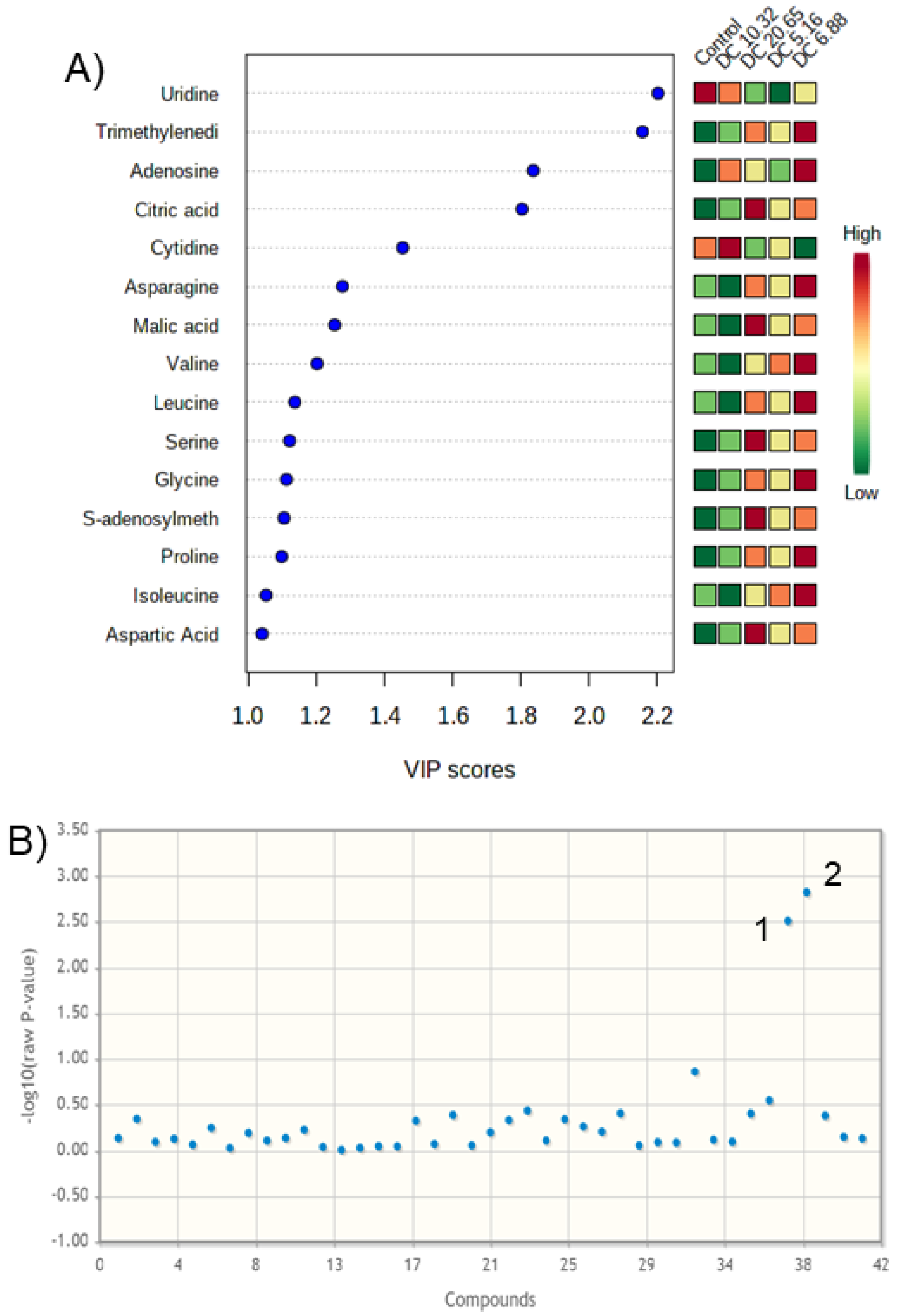

Figure S2. A) PLS-DA loadings figure demonstrates the variable importance in projection (VIP) and the relative concentration of the metabolites following DCAA exposure in each corresponding group of the study. Results demonstrated for the top 15 metabolites responsible for variance in the metabolome. **B**) ANOVA of metabolite concentrations, inosine (1) and guanosine (2), are statistically significant from the control group after exposure to DCAA ($p < 0.05$). Individual metabolite change figures can be found in Figure S4.

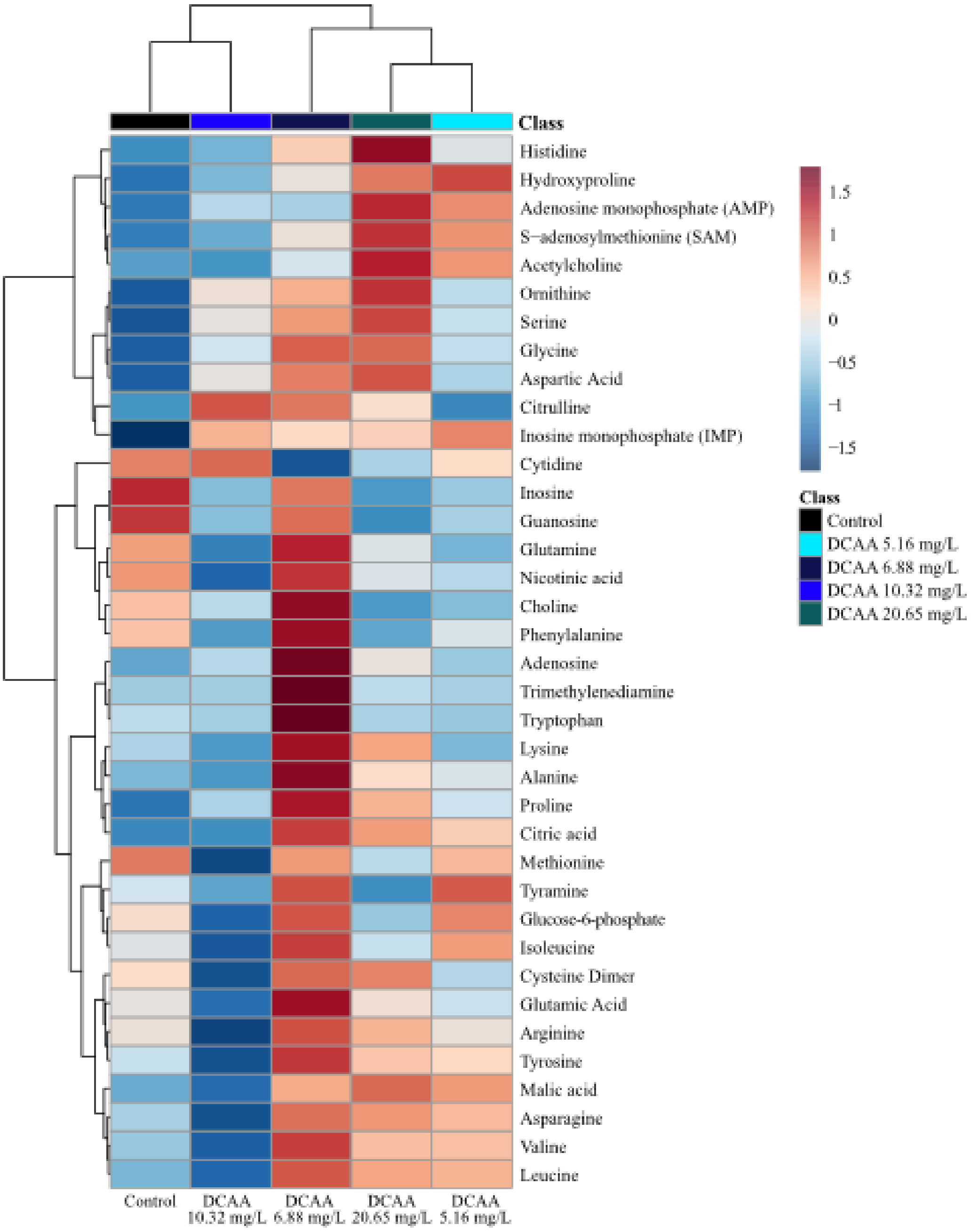

Figure S3. Analysis of averaged metabolite concentrations ($n = 12$) where 0 is the average metabolite concentrations across all treatment groups (DCAA exposure and the control). The colour gradient (blue to red) show the relative difference compared to the average metabolite concentration and correlated treatments and metabolites are linked on the upper x-axis and left y-axis respectively. .

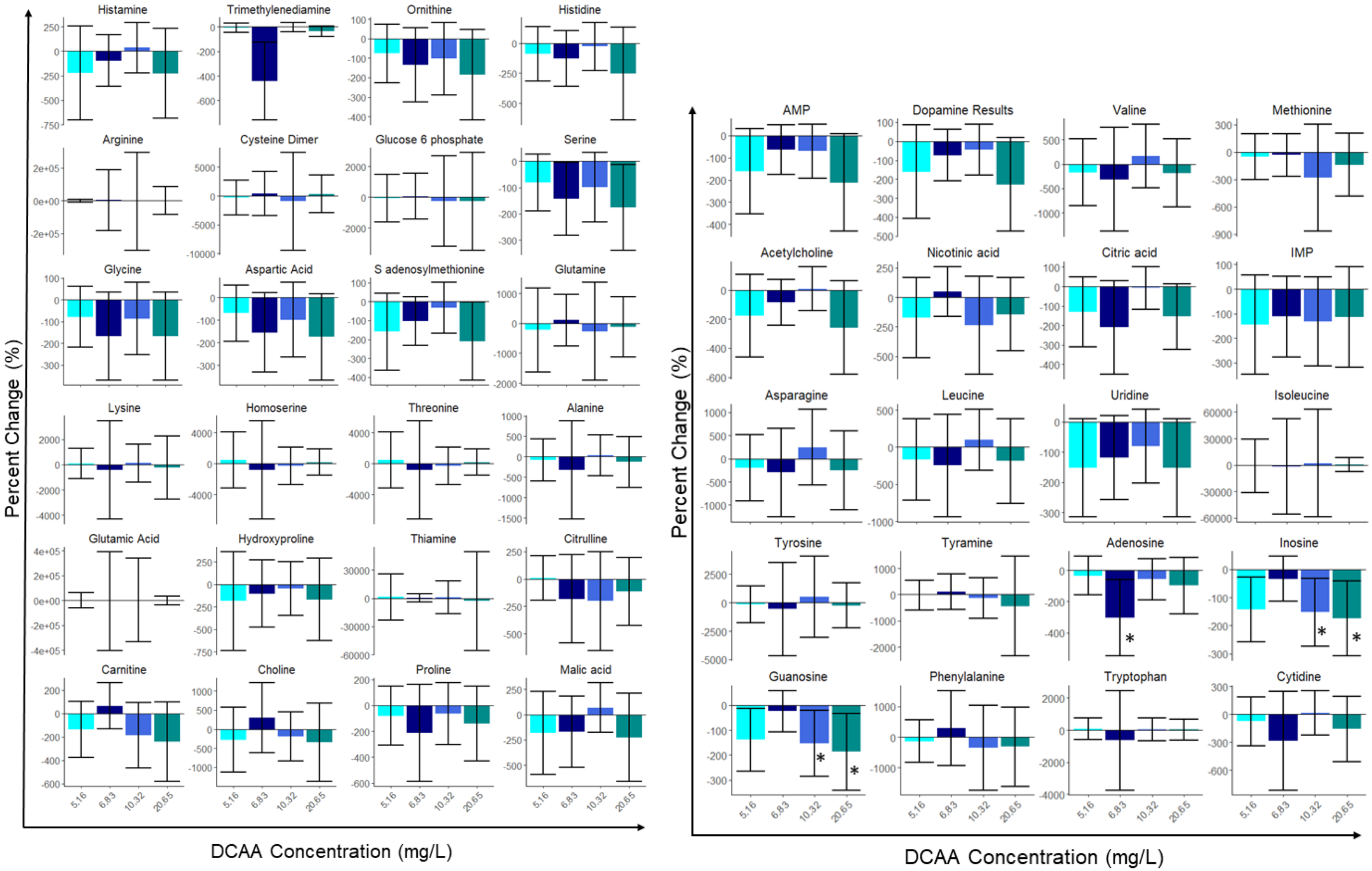

Figure S4. Averaged ($n = 12$) metabolite percent changes relative to the control. Statistical significance ($p < 0.05$) was determined with a two-tailed, equal variance t-test and is outlined with the presence of an asterisk (*).

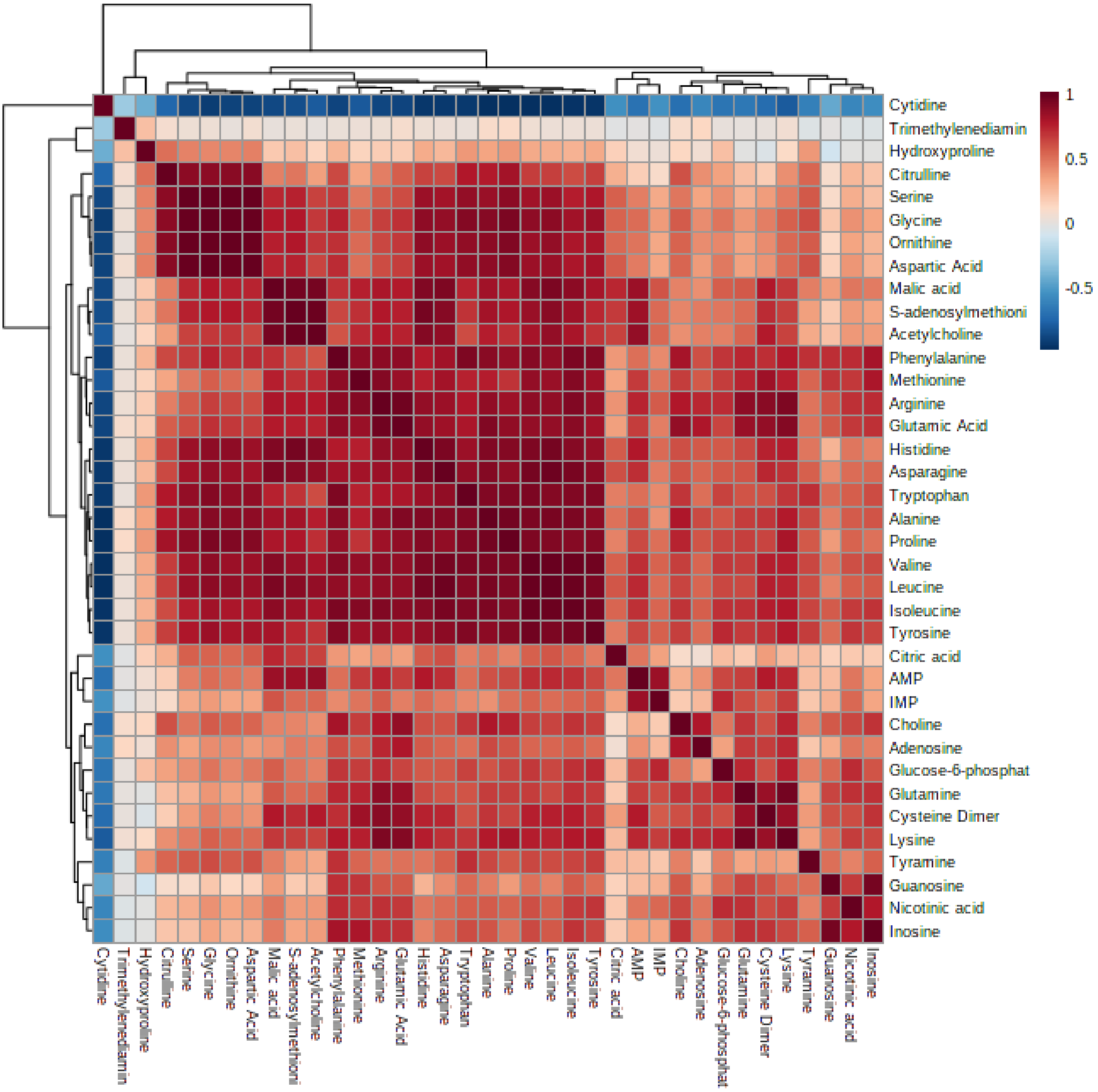

Figure S5. Pearson correlation plot for DCAA exposure which demonstrates the correlation between metabolites measured in *D. magna* extract. Dark red, indicates a strong positive correlation and dark blue represents a strong negative correlation between the two given metabolites.

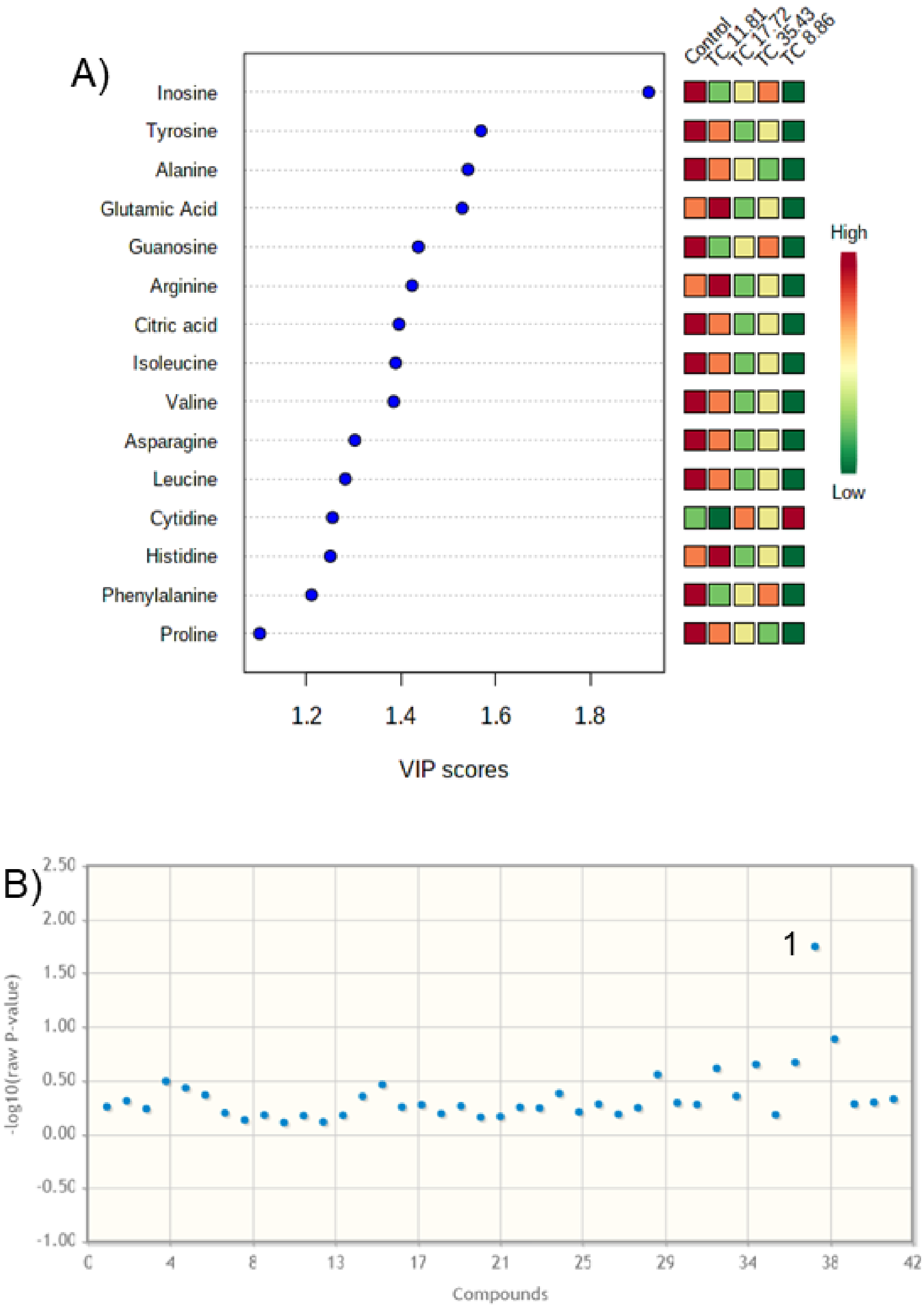

Figure S6. A) PLS-DA loadings figure demonstrates the variable importance in projection (VIP) and the relative concentration of the metabolites following TCAA exposure in each corresponding group of the study. Results demonstrated for the top 15 metabolites responsible for variance in the metabolome. B) ANOVA of metabolite concentrations, inosine (1) is statistically significant from the control group after exposure to TCAA ($p < 0.05$). Individual metabolite change figures can be found in Figure S8.

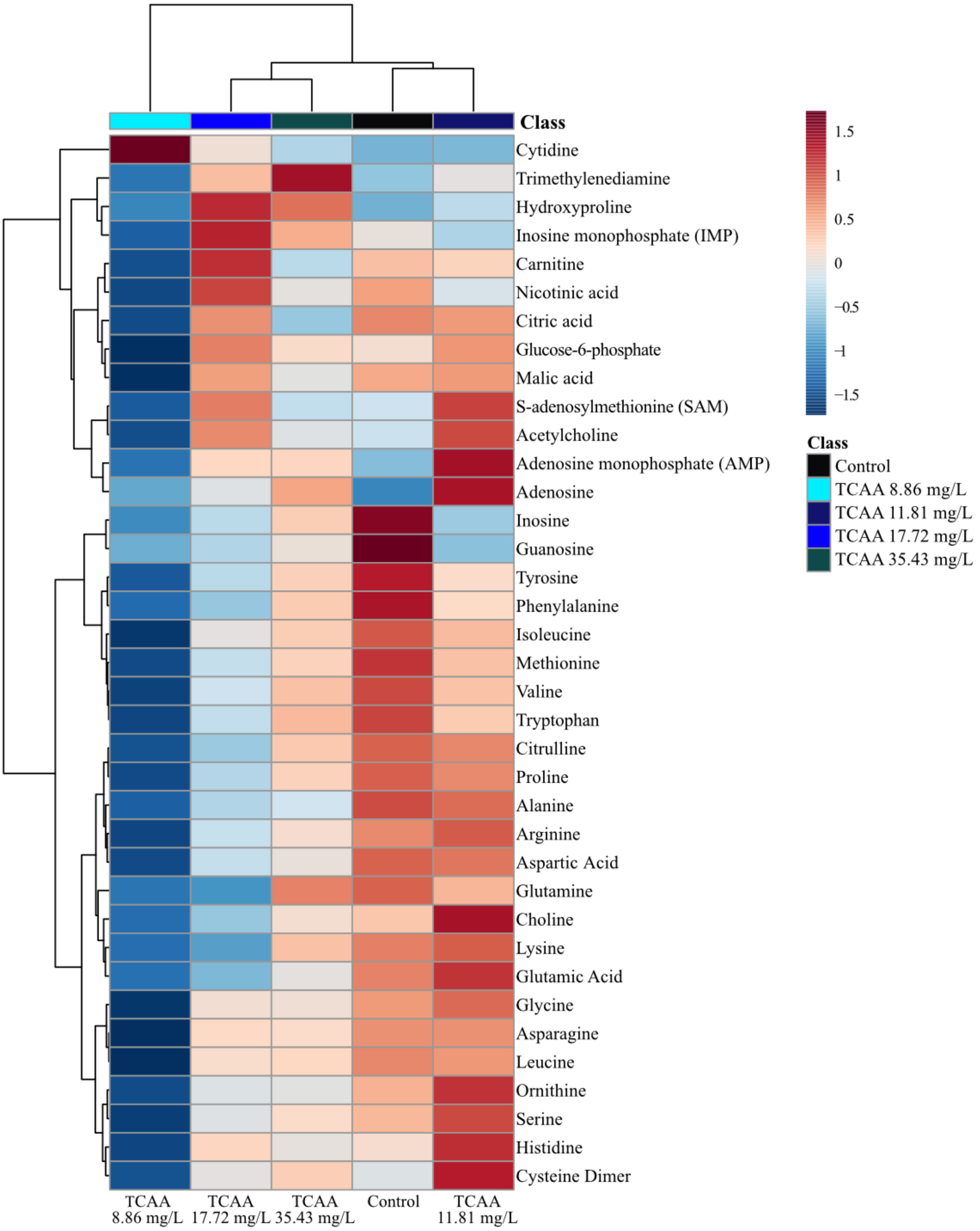

Figure S7. Analysis of averaged metabolite concentrations (*n* = *12*) where 0 is the average metabolite concentrations across all treatment groups (TCAA exposure and the control). The colour gradient (blue to red) show the relative difference compared to the average metabolite concentration and correlated treatments and metabolites are linked on the upper x-axis and left y-axis respectively.

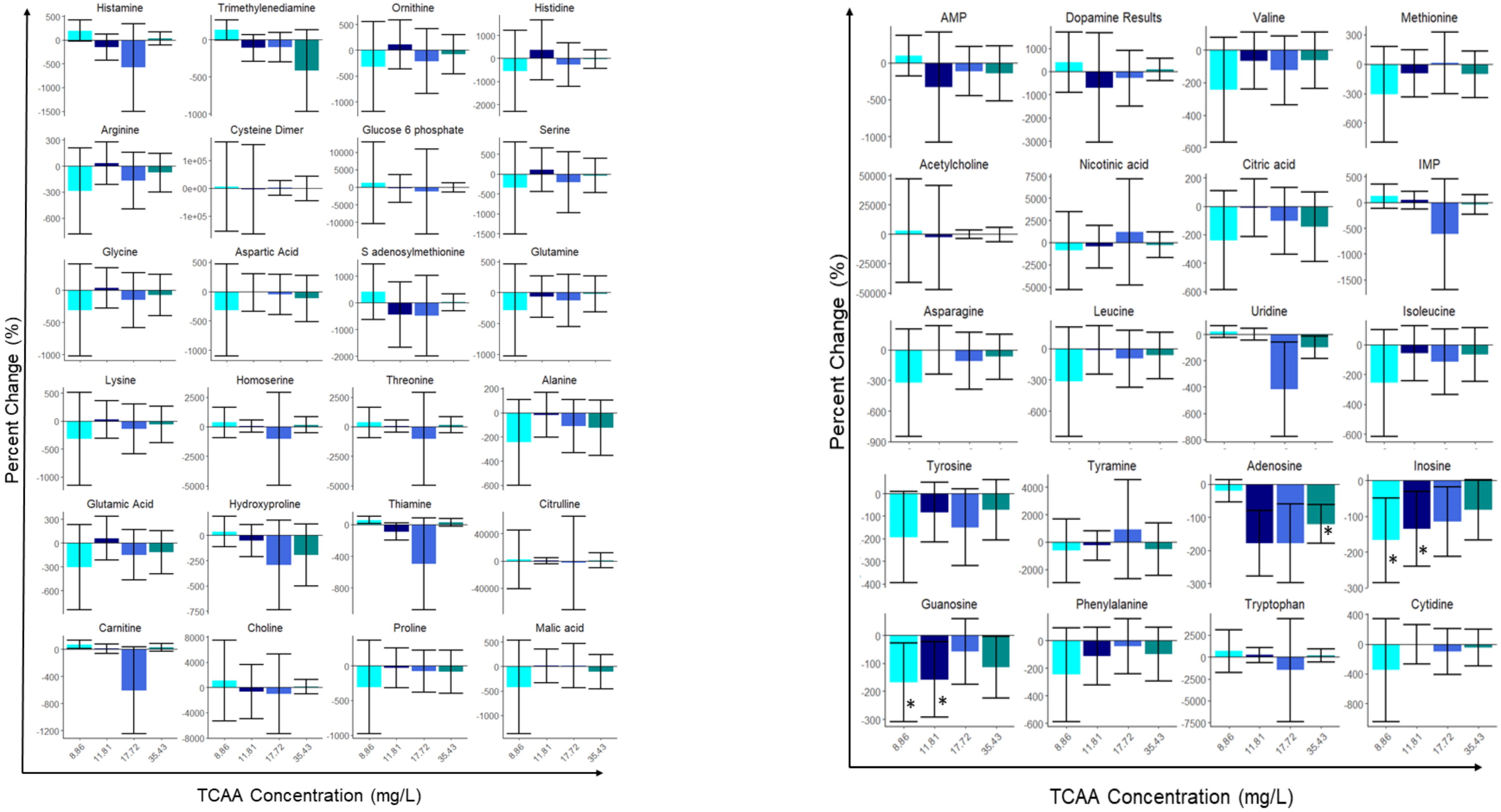

Figure S8. Averaged ($n = 12$) metabolite percent changes following sub-lethal exposure to TCAA relative to the control. Statistical significance ($p < 0.05$) was determined with a two-tailed, equal variance *t*-test and is outlined with the presence of an asterisk (*).

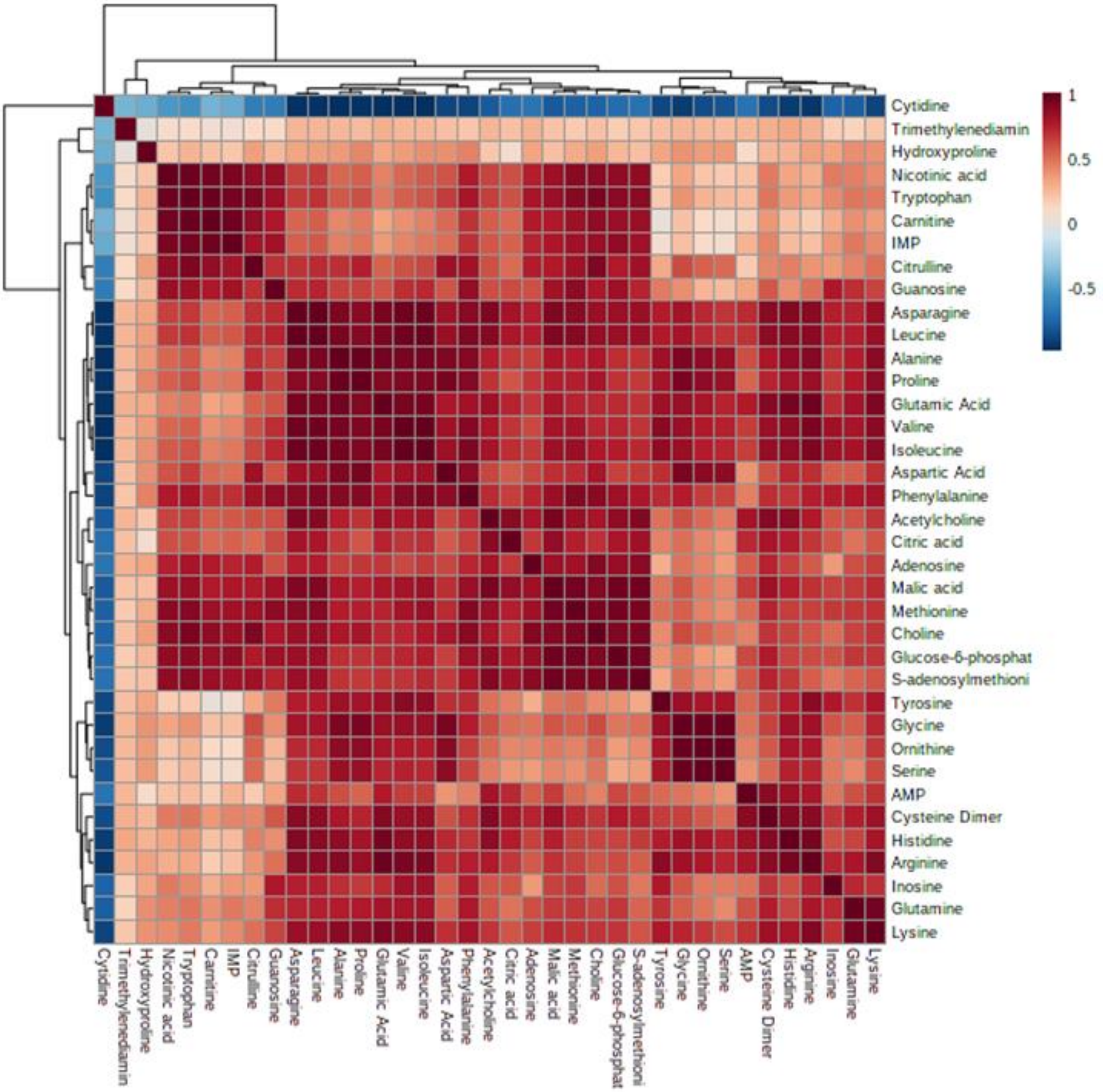

Figure S9. Pearson correlation plot for TCAA exposure which demonstrates the correlation between metabolites measured in *D. magna* extract. Dark red, indicates a strong positive correlation and dark blue represents a strong negative correlation between the two given metabolites.

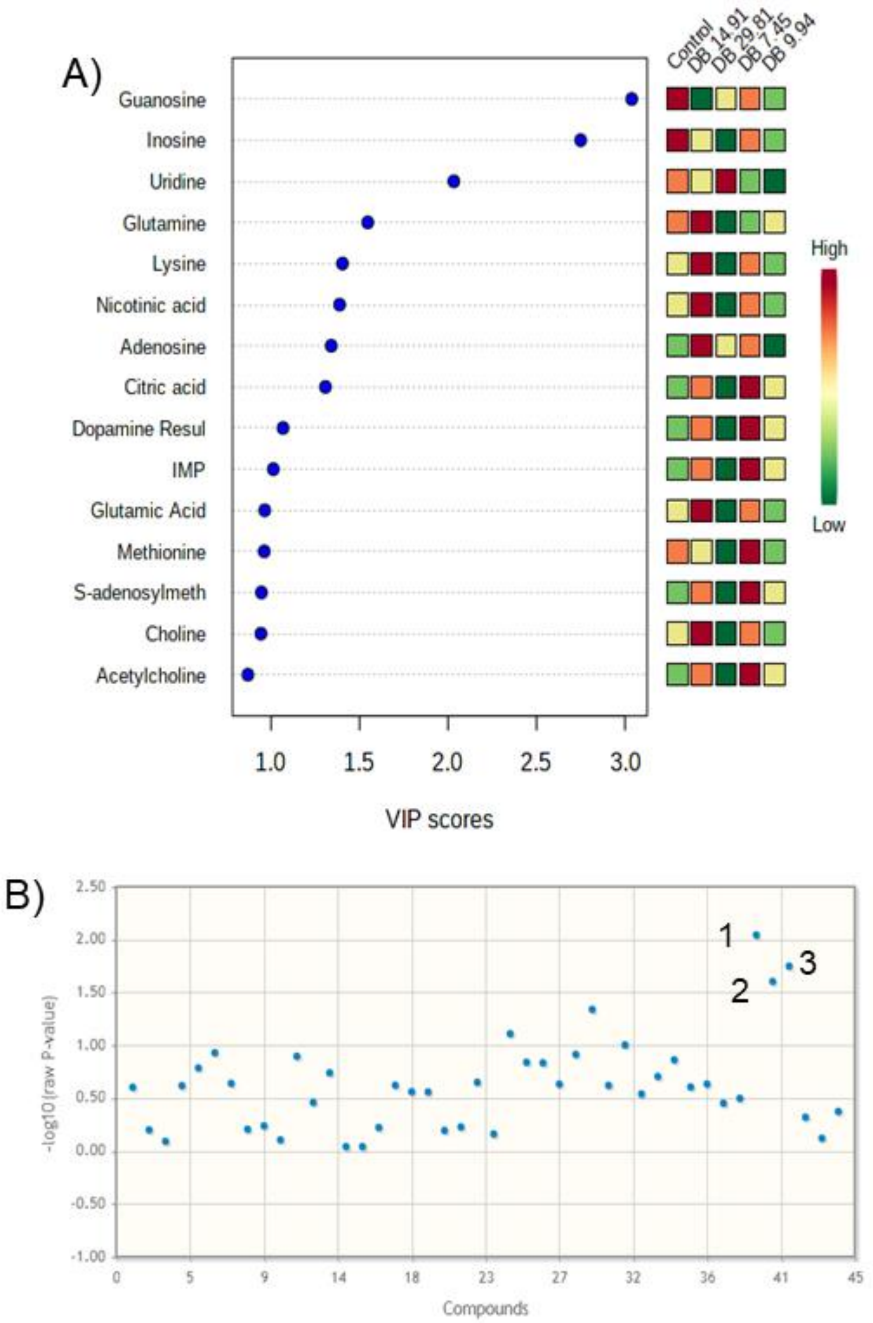

Figure S10. A) PLS-DA loadings figure demonstrates the variable importance in projection (VIP) and the relative concentration of the metabolites following DBAA exposure in each corresponding group of the study. Results demonstrated for the top 15 metabolites responsible for variance in the metabolome. B) ANOVA of metabolite concentrations, adenosine (1), inosine (2), and guanosine (3) is statistically significant from the control group after exposure to DBAA ($p < 0.05$). Individual metabolite change figures can be found in Figure S12.

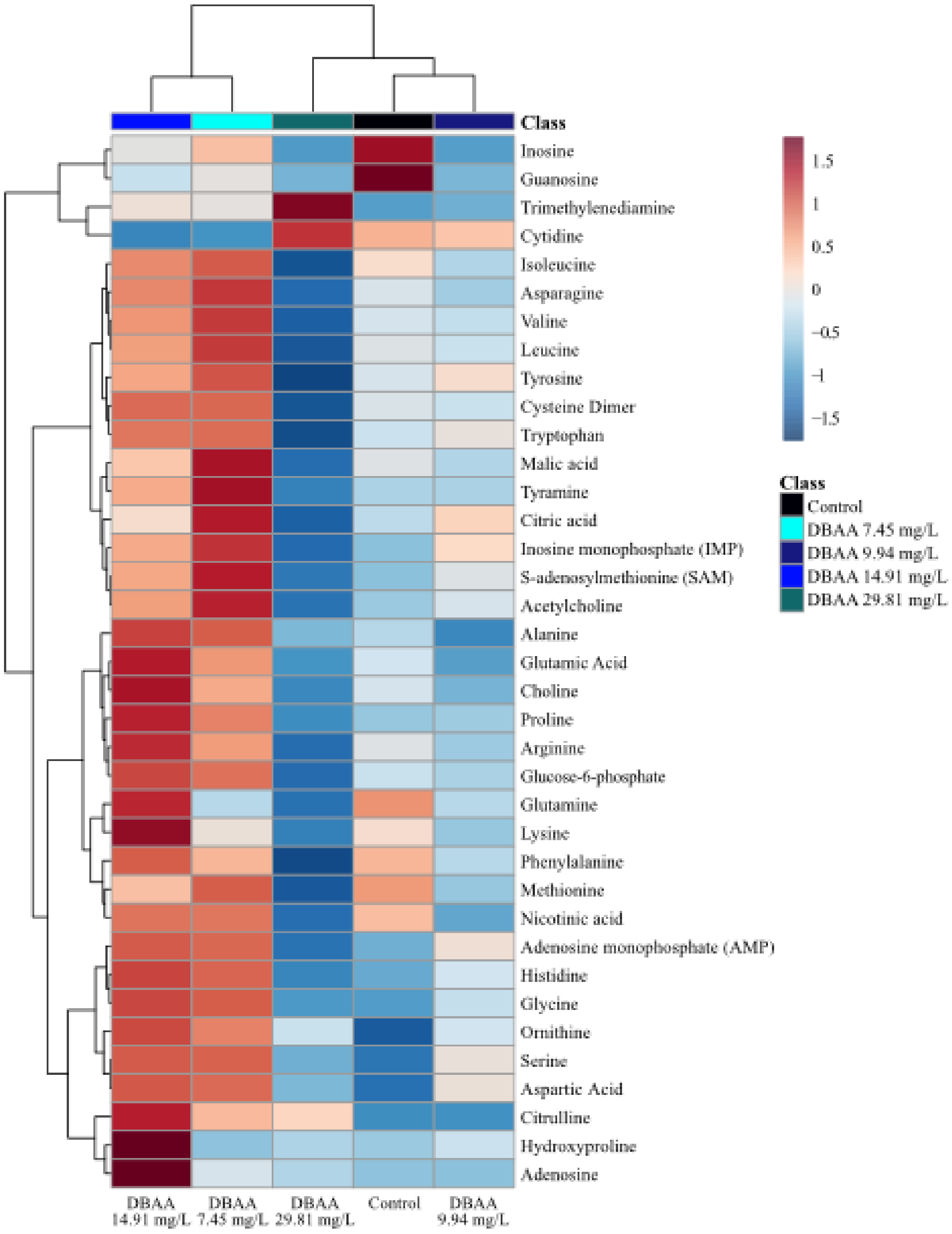

Figure S11. Analysis of averaged metabolite concentrations (*n* = 12) where 0 is the average metabolite concentrations across all treatment groups (DBAA exposure and the control). The colour gradient (blue to red) show the relative difference compared to the average metabolite concentration and correlated treatments and metabolites are linked on the upper x-axis and left y-axis respectively. .

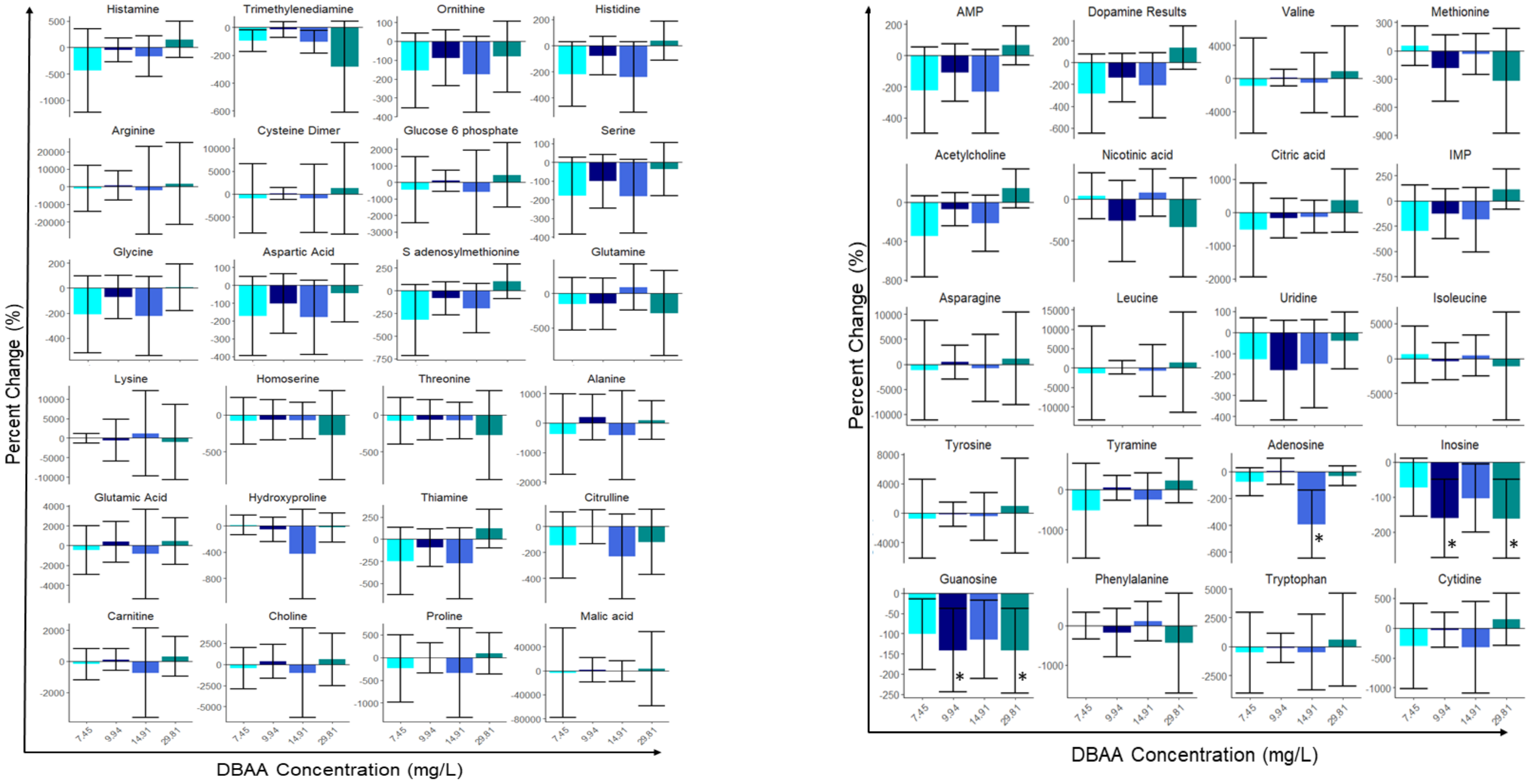

Figure S12. Averaged ($n = 12$) metabolite percent change following sub-lethal exposure to DBAA relative to the control. Statistical significance ($p < 0.05$) was determined with a two-tailed, equal variance *t*-test and is outlined with the presence of an asterisk (*).

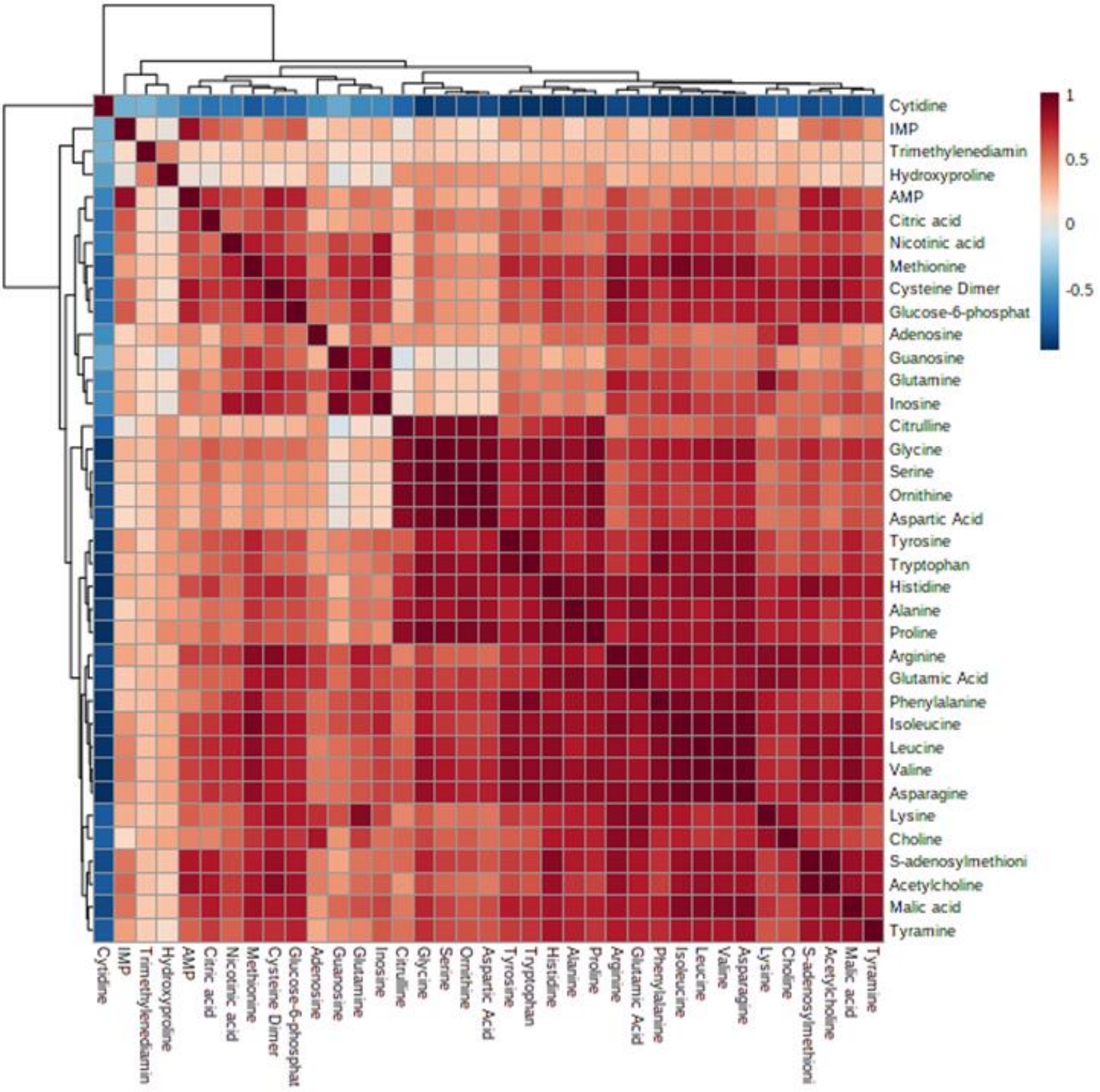

Figure S13. Pearson correlation plot for TCAA exposure which demonstrates the correlation between metabolites measured in *D. magna* extract. Dark red, indicates a strong positive correlation and dark blue represents a strong negative correlation between the two given metabolites.

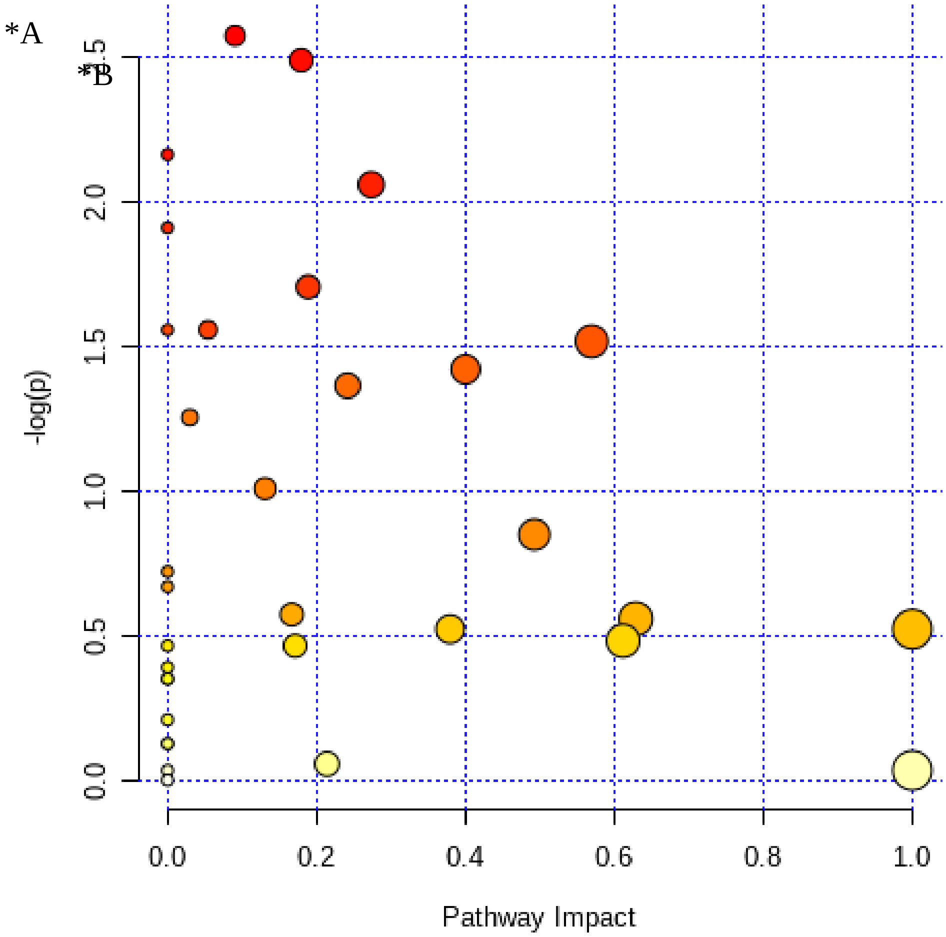

Figure S14. MetaboAnalyst pathway analysis for sub-lethal exposure of *D. magna* to DCAA at 20.65 mg/L. Statistical significance ($p < 0.10$) denoted asterisk (*); (A) Citrate Cycle (TCA cycle), (B) Purine metabolism.

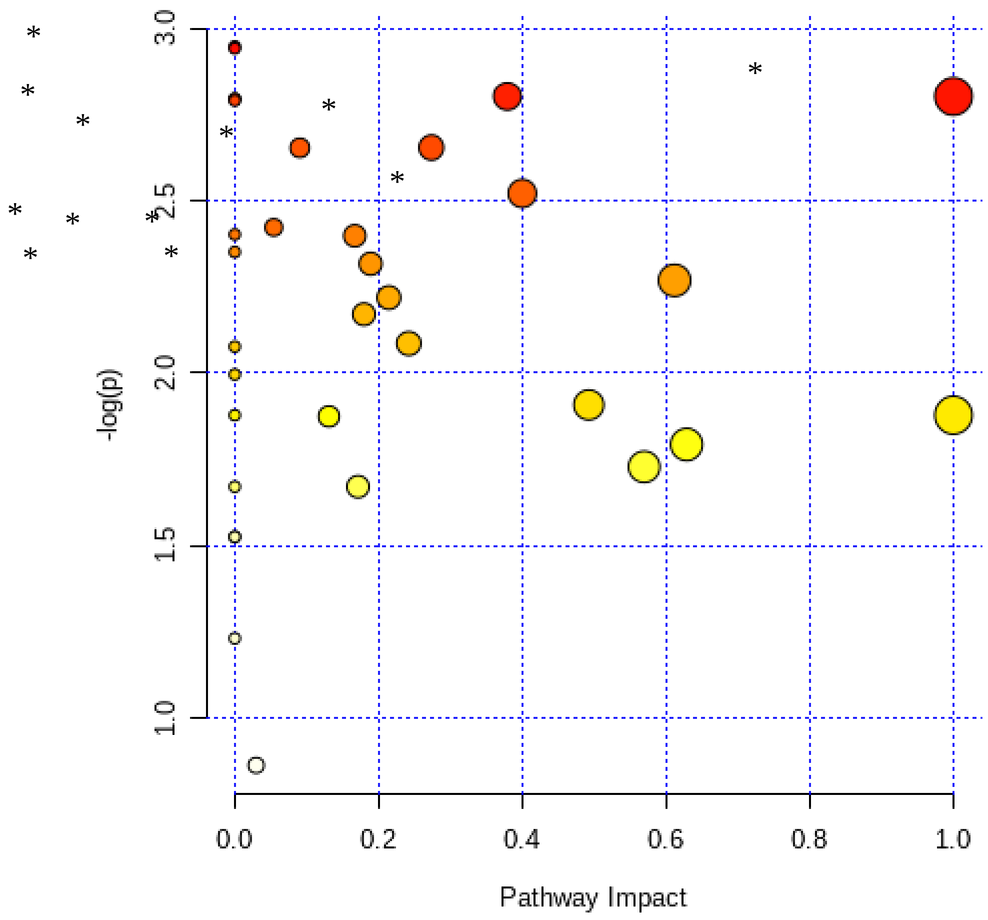

Figure S15. MetaboAnalyst pathway analysis for sub-lethal exposure of *D. magna* to TCAA at 8.86 mg/L. Statistical significance (*p* < *0.10*) denoted by asterisk (*). Impacted pathways listed in Table 2 in main article.

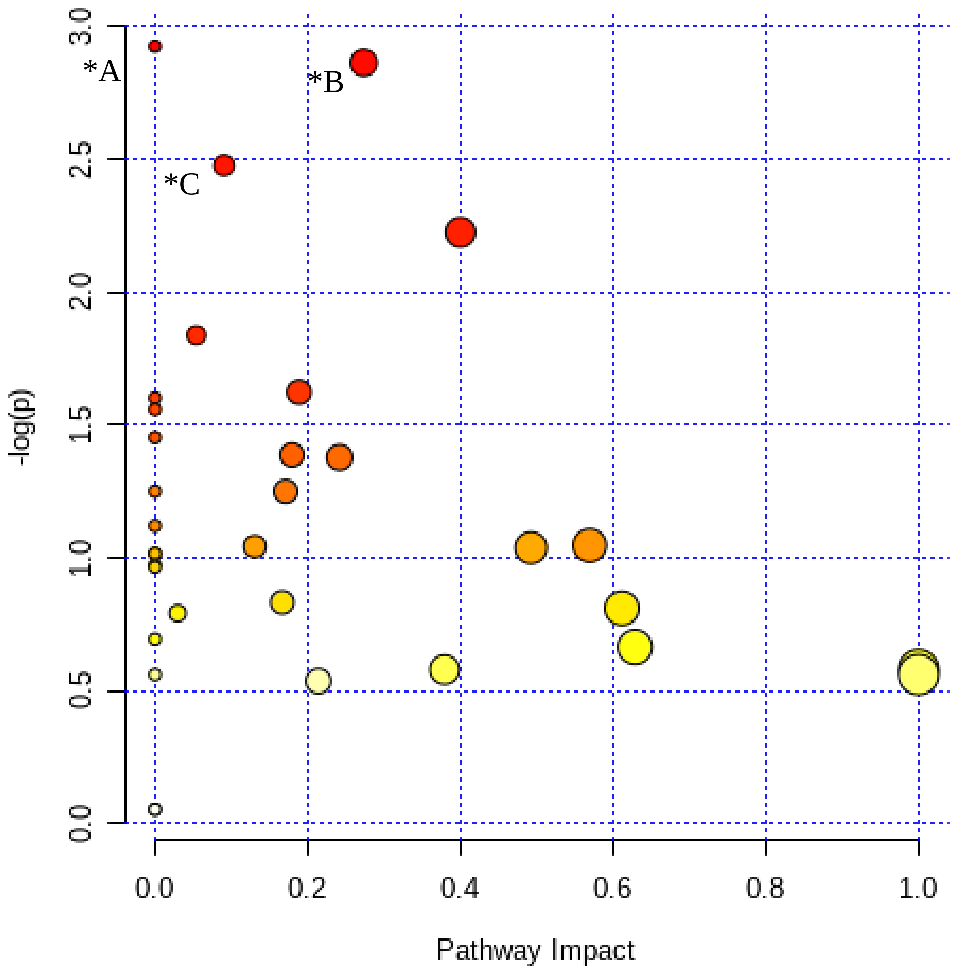

Figure S16. MetaboAnalyst pathway analysis for sub-lethal exposure of *D. magna* to DBAA at 7.45 mg/L. Statistical significance ($p < 0.10$) denoted by asterisk (*); (A) Thiamine metabolism, (B) Tyrosine metabolism, (C) Citrate cycle (TCA cycle).

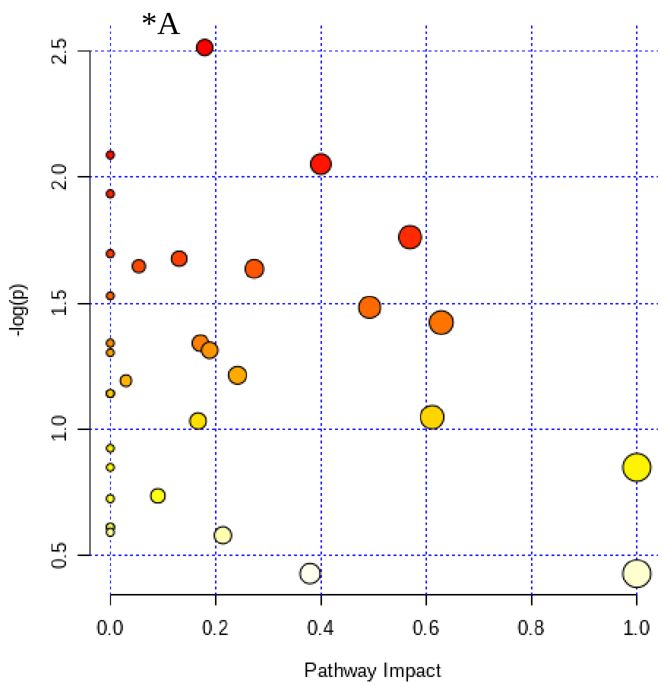

Figure S17. MetaboAnalyst pathway analysis for sub-lethal exposure of *D. magna* to DBAA at 7.45 mg/L. Statistical significance ($p < 0.10$) denoted by asterisk (*); (A) Purine metabolism.

References

85. Xie, Y. Analyzing Haloacetic Acids Using Gas Chromatography/Mass Spectrometry. *Water Res.* **2001**, *35*, 1599–1602, doi:10.1016/S0043-1354(00)00397-3.
86. Meng, L.; Wu, S.; Ma, F.; Jia, A.; Hu, J. Trace determination of nine haloacetic acids in drinking water by liquid chromatography–electrospray tandem mass spectrometry. *J. Chromatogr. A* **2010**, *1217*, 4873–4876, doi:10.1016/J.CHROMA.2010.04.074.
87. Luo, Q.; Wang, D.; Wei, Z.; Wang, Z. Optimized chromatographic conditions for separation of halogenated acetic acids by ultra-performance liquid chromatography-electrospray ionization-mass spectrometry. *J. Chromatogr. A* **2013**, *1277*, 26–34, doi:10.1016/j.chroma.2012.12.046.
